# Impact of Dendritic Size and Dendritic Topology on Burst Firing in Pyramidal Cells

**DOI:** 10.1371/journal.pcbi.1000781

**Published:** 2010-05-13

**Authors:** Ronald A. J. van Elburg, Arjen van Ooyen

**Affiliations:** 1 Department of Artificial Intelligence, Faculty of Mathematics and Natural Sciences, University of Groningen, Groningen, The Netherlands; 2 Department of Integrative Neurophysiology, Center for Neurogenomics and Cognitive Research, VU University Amsterdam, Amsterdam, The Netherlands; Université Paris Descartes, Centre National de la Recherche Scientifique, France

## Abstract

Neurons display a wide range of intrinsic firing patterns. A particularly relevant pattern for neuronal signaling and synaptic plasticity is burst firing, the generation of clusters of action potentials with short interspike intervals. Besides ion-channel composition, dendritic morphology appears to be an important factor modulating firing pattern. However, the underlying mechanisms are poorly understood, and the impact of morphology on burst firing remains insufficiently known. Dendritic morphology is not fixed but can undergo significant changes in many pathological conditions. Using computational models of neocortical pyramidal cells, we here show that not only the total length of the apical dendrite but also the topological structure of its branching pattern markedly influences inter- and intraburst spike intervals and even determines whether or not a cell exhibits burst firing. We found that there is only a range of dendritic sizes that supports burst firing, and that this range is modulated by dendritic topology. Either reducing or enlarging the dendritic tree, or merely modifying its topological structure without changing total dendritic length, can transform a cell's firing pattern from bursting to tonic firing. Interestingly, the results are largely independent of whether the cells are stimulated by current injection at the soma or by synapses distributed over the dendritic tree. By means of a novel measure called mean electrotonic path length, we show that the influence of dendritic morphology on burst firing is attributable to the effect both dendritic size and dendritic topology have, not on somatic input conductance, but on the average spatial extent of the dendritic tree and the spatiotemporal dynamics of the dendritic membrane potential. Our results suggest that alterations in size or topology of pyramidal cell morphology, such as observed in Alzheimer's disease, mental retardation, epilepsy, and chronic stress, could change neuronal burst firing and thus ultimately affect information processing and cognition.

## Author Summary

Neurons possess highly branched extensions, called dendrites, which form characteristic tree-like structures. The morphology of these dendritic arborizations can undergo significant changes in many pathological conditions. It is still poorly known, however, how alterations in dendritic morphology affect neuronal activity. Using computational models of pyramidal cells, we study the influence of dendritic tree size and branching structure on burst firing. Burst firing is the generation of two or more action potentials in close succession, a form of neuronal activity that is critically involved in neuronal signaling and synaptic plasticity. We found that there is only a range of dendritic tree sizes that supports burst firing, and that this range is modulated by the branching structure of the tree. We show that shortening as well as lengthening the dendritic tree, or even just modifying the pattern in which the branches in the tree are connected, can shift the cell's firing pattern from bursting to tonic firing, as a consequence of changes in the spatiotemporal dynamics of the dendritic membrane potential. Our results suggest that alterations in pyramidal cell morphology could, via their effect on burst firing, ultimately affect cognition.

## Introduction

Neurons exhibit a wide range of intrinsic firing patterns with respect to both spike frequency and spike pattern [1]–[3]. A distinct type of firing pattern that is critically involved in neuronal signaling and synaptic plasticity is burst firing, the generation of clusters of spikes with short interspike intervals [4]. Bursts can improve the signal-to-noise ratio of neuronal responses [5] and may convey specific stimulus-related information [6]. Bursts of spikes can be more effective than single spikes in inducing synaptic long-term potentiation (LTP) [7], [8], or can even determine whether LTP or LTD (long-term depression) occurs [9]. In synapses with short-term facilitation, bursts can be transmitted more reliably than isolated spikes [10].

Electrophysiology, in combination with computational modeling, has elucidated the ionic mechanisms underlying intrinsic neuronal burst firing. Two main classes of mechanisms have been distinguished [4]. In so-called dendrite-independent mechanisms—responsible for bursting in thalamic relay neurons [11], for example—the fast, spike-generating conductances and the slow, burst-controlling conductances are co-localized in the soma. Conversely, in dendrite-dependent mechanisms—involved in pyramidal cell burst firing—these conductances are distributed across the soma and dendrites, with the interaction between somatic and dendritic conductances playing an essential role in burst generation. Dendritic voltage-gated Na^+^ and K^+^ channels, which promote propagation of action potentials from the soma into the dendrites, cause the dendrites to be depolarized when, at the end of a somatic spike, the soma is hyperpolarized, leading to a return current from dendrites to soma. The return current gives rise to a depolarizing afterpotential at the soma, which, if strong enough, produces another somatic spike [12], [13]. This whole process was described by Wang [13] as ‘ping-pong’ interaction between soma and dendrites.

Although ion channels play a pivotal role in burst firing, dendritic morphology also appears to be an important factor. In many cell types, including neocortical and hippocampal pyramidal cells [14]–[17], neuronal firing patterns and the occurrence of bursts are correlated with dendritic morphology. Results from modeling studies also suggest a relationship between dendritic morphology and firing pattern [18]–[21]. However, these studies are mainly correlative [21], focus on morphologically very distinct cell classes [18], use only the physiologically less appropriate stimulation protocol of somatic current injection, and do not investigate the impact of topological structure of dendritic arborizations. Consequently, the effects of dendritic size and dendritic topology on burst firing, and the underlying mechanisms, remain poorly known.

Considering that dendritic morphology can undergo significant changes in many pathological conditions, such as Alzheimer [22], [23], mental retardation [24], [25], epilepsy [26], and chronic stress [27]–[29], it is important to examine the implications of altered morphology for neuronal firing. Using computational models of neocortical pyramidal neurons, we here explore in a systematic and rigorous way the impact of a cell's dendritic morphology on the ping-pong mechanism of burst firing, under either somatic current injection or synaptic stimulation of the apical dendritic tree. Importantly, we thereby distinguish between the effects of size and topology of the apical dendrite. Furthermore, we identify the underlying mechanism by which morphology affects burst firing in the model.

## Methods

### Pyramidal cell

**Figure 1:**
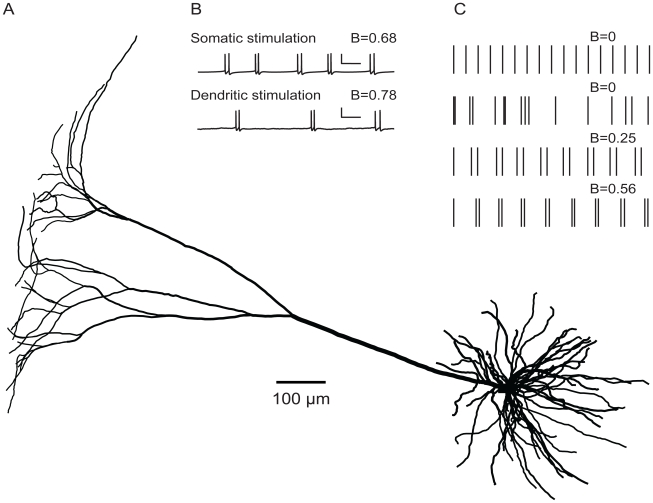
Pyramidal cell and burst firing. **A**, Reconstructed layer 5 pyramidal cell from cat visual cortex (adapted from [18]). Scale bar: 100 µm. The apical tree has a total length of 36865 µm, a total surface area of 25828 µm^2^, a total volume of 13292 µm^3^, a root segment with a diameter of 8.5 µm, and 41 terminal segments with diameters in the range 0.30–1.33 µm. The 10 basal dendrites have a total length of 10232 µm, a total surface area of 27396 µm^2^, a total volume of 7650 µm^3^, root segments with diameters in the range 1–4 µm, and in total 35 terminal segments with diameters in the range 0.59–1.31 µm. The MEP value (in units of the electrotonic length constant) of the apical dendrite is 0.74. The input conductance of the cell without basal dendrites is 7.9 µS and with basal dendrites 19.9 µS. **B**, Firing pattern (voltage trace) evoked in the cell of A by a continuous current injection at the soma (somatic stimulation) or by random activation of excitatory synapses distributed across the apical dendritic tree (dendritic stimulation). The burst measure *B* quantifies the degree of bursting. Scale bar: 100 ms, 50 mV. **C**, Examples of spike patterns illustrating different values of *B*. From top to bottom: a perfectly regular pattern without bursts, a random (Poisson) pattern, and two different burst patterns. The *B* value of both the regular and the Poisson spike train is zero, indicating complete lack of bursting. The two spike patterns with bursting illustrate that the higher the ratio of inter- to intraburst interspike intervals, the higher the value of *B*.

We use a morphologically and biophysically realistic model of a bursting layer 5 pyramidal cell from cat visual cortex (Fig. 1A) that is based on [18] (which in turn builds upon [30]). The model is implemented in NEURON [31] and captures the general features of bursting in pyramidal cells, particularly the interaction between soma and dendrites in burst generation [12], [13]. In the soma, the voltage-dependent currents and associated maximal conductances (in pS µm^−2^) are as follows: a fast sodium current, 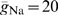; a slow voltage-dependent non-inactivating potassium current, 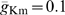; a fast non-inactivating potassium current, 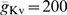; a slow calcium-activated potassium current, 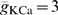; and a high voltage-activated calcium current, 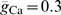. In the axon hillock, 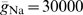 and 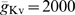 In the apical dendrite, the conductances are as in the soma, except that 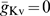. In both the soma and the dendrites, the membrane capacitance 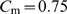 µF cm^−2^, the axial resistance 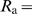
80 Ω cm, and 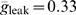. Internal calcium concentration is computed using entry via the high-voltage activated calcium current and removal by a first order pump, where the baseline calcium concentration is 0.1 µM and the time constant of calcium removal is 200 ms [18], [32]. The reversal potentials (in mV) are 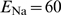, 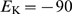, 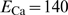, and 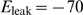. All currents are calculated using conventional Hodgkin-Huxley-style kinetics. For the specific rate functions for each current, we refer to [18].

The pyramidal cell is activated by either somatic or dendritic stimulation. For somatic stimulation, the cell is continuously stimulated with a fixed current injection of 0.2 nA at the soma. For dendritic stimulation, the cell is stimulated by synapses that are regularly distributed across the apical dendrite, with a density of 1 synapse per 20 µm^2^. For this synaptic density, the total input current, based on the current transfer at a single synapse, is approximately the same as with somatic stimulation.

The excitatory synaptic input is mediated by AMPA receptors. The time course of conductance changes follows an alpha function 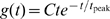, where 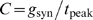, with the peak time 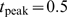 ms, the peak conductance 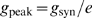, 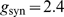 nS, and the reversal potential 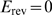 mV [33]. Each synapse is randomly activated, whereby the time intervals between the activations of a synapse are drawn from a negative exponential distribution 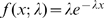, where 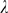 is the mean of the distribution. Over the time period of a complete simulation, this results in a Poisson distribution of synaptic activation times. For each synapse, the mean activation frequency 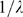 is set to 1 Hz, and each synapse is activated independently of the other synapses.

The firing patterns were recorded from the soma. Each simulation lasted 10000 ms, of which the first 1000 ms were discarded in the analysis in order to remove possible transient firing patterns.

**Figure 2:**
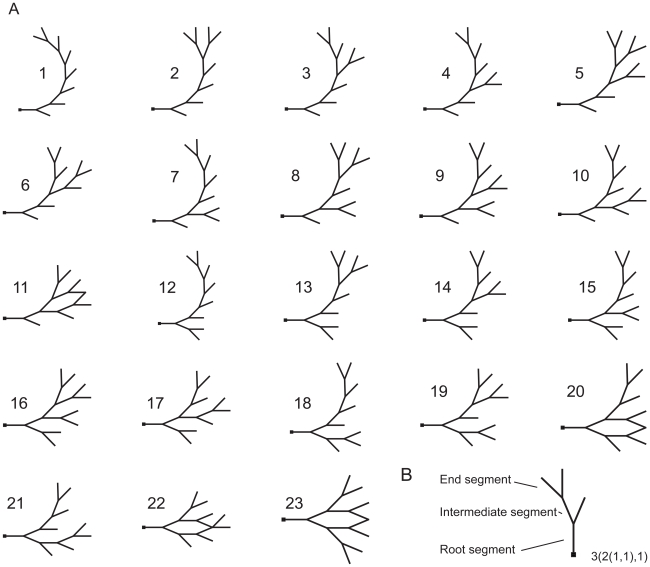
Morphologically simplified cells. **A**, The set of 23 neurons consisting of all the topologically different trees with 8 terminal segments. The neurons are ordered according to the degree of symmetry of their branching structure (see Methods), with 1 having the most asymmetric and 23 the most symmetric tree. **B**, In the description of the morphology of a dendritic tree, we distinguish between terminal or end segments (which terminate in a tip) and intermediate segments (which terminate in a branch point). The root segment is the intermediate segment that is connected to the soma. The connectivity pattern of the segments is called the topology of a tree. At the right is the description of this tree in the notation we employed to order the trees.

In studying the influence of pyramidal cell morphology on burst firing, we distinguish between dendritic size and dendritic topology. The size of a dendritic tree is the total length of all its dendritic segments. The segment between the soma and the first branch point is called the root segment (see Fig. 2B). Dendritic segments between two branch points are intermediate segments, and segments between a branch point and a terminal tip are terminal segments. The topology of a dendritic tree is the way in which the dendritic segments are connected to each other. For example, a tree with a given number of terminal segments can be connected in a fully asymmetrical or a fully symmetrical way (see Fig. 2B, dendritic trees 1 and 23, respectively).

To investigate how the dendritic size of the pyramidal cell influences burst firing, we varied the total length of the cell's apical dendrite according to two methods. In the first method, we successively pruned terminal segments from the apical dendritic tree. Starting with the full pyramidal cell morphology, in each round of pruning we randomly removed a number of terminal segments from the apical dendritic tree. Each terminal segment had a chance of 0.3 to be removed. From the reduced dendritic tree, we again randomly cut terminal segments, and so on, until in principle the whole apical dendrite was eliminated. This whole procedure was repeated 20 times. The density of synapses was kept constant during pruning, so with dendritic stimulation pruning also changed the total input to the cell. With somatic simulation, the total input to the cell did not change when the apical dendrite was pruned.

In the second method, we kept the dendritic arborization intact and changed the size of the apical dendrite by multiplying the lengths of all its segments by the same factor. Thus in this way the entire apical dendritic tree was compressed or expanded. For dendritic stimulation, we kept the total synaptic input to the cell constant by adapting the density of the synapses. So, both with somatic and dendritic stimulation, the total input to the cell did not change when the size of the apical dendrite was modified.

To examine the impact of the cell's dendritic branching structure on burst firing, we varied the topology of the apical dendritic tree by swapping branches within the tree. The apical dendritic trees that were generated in this way all have exactly the same total dendritic length and other metrical properties such as total dendritic surface area and differ only in their topological structure. The total input to the cell, both with somatic and dendritic stimulation, did not change when the topological structure was altered.

### Morphologically simplified cells

To facilitate a systematic analysis of the role of dendritic size and dendritic topology in shaping burst firing, we also use a set of morphologically simplified neurons. The neurophysiological complexity of these neurons is similar to that of the full pyramidal cell model. For a systematic study, one must use trees with a relatively small number of terminal segments, because otherwise the number of topologically different trees becomes so large that simulating all of them becomes impossible. For a tree with only 12 terminal segments, for example, there already exist as many as 451 different tree topologies [34]. Here, we use a set of 23 neurons consisting of all the topologically different trees with 8 terminal segments (Fig. 2). The trees may also be thought of as representing the backbones of potentially much larger dendritic arborizations.

All segments in the tree (intermediate and terminal segments; see Fig. 2B) have the same length, so that the different tree topologies do not differ in total dendritic length. In almost all types of neurons, including neocortical pyramidal cells, the diameters of dendritic segments decrease at each branch point, with terminal segments having the smallest diameter [35], [36]. In the trees we use, the diameter of a parent segment, 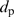, is related to the diameters of its daughter segments, 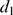 and 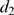, as 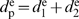, where the branch power *e* is equal to 1.5 (Rall's power law) [37]. For pyramidal cells, values of the branch power were found to range between 1.5 and 2 [35], [36]. Since terminal segment diameters show only a narrow range of values [38], all terminal segments were given the same diameter (0.7 µm) [35], [38], while the diameters of intermediate segments were calculated using Rall's power law. This implies that asymmetrical topologies will have a higher total dendritic surface area than symmetrical topologies. We therefore also considered the case in which all segments in the tree have the same diameter (3 µm), so that the different tree topologies do not differ in dendritic surface area. All neurons also have a soma compartment, with a diameter and length of 14 µm [39].

The ion channel types and densities are based on Mainen and Sejnowski's [18] reduced model. In the soma, there is a fast sodium current, 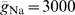, and a fast non-inactivating potassium current, 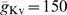 (maximal conductances, in pS µm^−2^). The dendrites contain a fast sodium current, 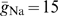; a slow voltage-dependent non-inactivating potassium current, 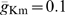; a slow calcium-activated potassium current, 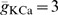; a high voltage-activated calcium current, 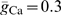; and a leak current, 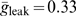. Internal calcium concentration is computed using entry via the high-voltage activated calcium current and removal by a first order pump, where the baseline calcium concentration is 0.1 µM and the time constant of calcium removal is 200 ms [18], [32]. For both the soma and the dendrites, the membrane capacitance 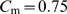 µF cm^−2^ and the axial resistance 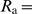
80 Ω cm. The reversal potentials (in mV) are 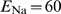, 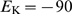, 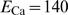, and 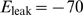. All currents are calculated using conventional Hodgkin-Huxley-style kinetics. For the specific rate functions for each current, we refer to [18].

As in the pyramidal cell model with full morphological complexity, the neurons are activated by either somatic or dendritic stimulation. All the tree topologies receive the same input. For somatic stimulation, the neurons are continuously stimulated with a fixed current injection of 0.03 nA (0.1 nA for the non-Rall neurons, in which segment diameter is equal throughout the dendritic tree). For dendritic stimulation, the cells are stimulated by 600 synapses, with on each terminal or intermediate segment (in total 15 segments for a tree with 8 terminal segments) 40 uniformly distributed synapses. With this number of synapses, the total input current, based on the current transfer at a single synapse, is approximately the same as with somatic stimulation. The synaptic input is mediated by AMPA receptors, with the same parameters as in the full pyramidal cell model. Also as in the full pyramidal cell model, each synapse is randomly activated according to a Poisson process, with a mean activation frequency of 1 Hz.

The simulations were performed in NEURON [31]. The firing patterns were recorded from the axosomatic compartment. Each simulation lasted 10000 ms, of which the first 1000 ms were discarded in the analysis in order to remove possible transient firing patterns.

To examine how the size of the dendritic tree influences firing pattern, we changed the total dendritic length of a given tree topology by multiplying the lengths of all its segments by the same factor. For dendritic stimulation, the number of synapses on the tree was thereby kept constant. So, both with somatic and dendritic stimulation, the total input to the cell did not change when the size of the dendritic tree was modified.

For presenting the firing patterns from the different tree topologies, we ordered the trees according to the degree of symmetry of their branching structure. To do this, we used a variant of the ranking scheme proposed by Harding [34], [40]. A binary tree can be described by denoting at each node, i.e., a branch point or the root, the sizes of the subtrees (in number of terminal segments) it carries. For example, a tree of size 1 is simply denoted as 1. A tree of size 2 is denoted as 2(1,1). For trees of size 3 (see Fig. 2B), there are two possibilities, 3(1,2(1,1)) and 3(2(1,1),1), but they do not represent topologically different trees, because the only difference is the order of the two subtrees. For trees with four terminal segments, there are two topologically different trees, 4(2(1,1),2(1,1)) and 4(3(2(1,1),1),1). Note that the last tree can also be written in several other ways, e.g., 4(3(1,2(1,1)),1).

To obtain a unique notation, we applied the following two rules. First, if the subtrees at a particular node have a different size, the largest subtree is put left of the comma. So we write 3(2(1,1),1) instead of 3(1,2(1,1)). Second, to order (sub)trees of equal size, we consider to be larger the tree that has the highest number at the first figure in which the tree descriptions differ. So of the following two trees, 4(2(1,1),2(1,1)) and 4(3(2,1),1), the second one is considered to be larger (since 3>2). Thus, in a description of an 8-terminal tree of which these two trees are the subtrees, the second subtree is put first, i.e., 8(4(3(2,1),1),4(2(1,1),2(1,1))).

Once all tree topologies were written in a unique form, they were ordered according to their size (in the extended sense, as described above), whereby the largest one was put first in the list. Since two trees can now be ordered simply by looking at the first figure in which their descriptions differ, this ordering is called a reverse lexicographical ordering. Applied to trees of the same size, it puts trees in order of symmetry, with the most asymmetrical tree first and the most symmetrical tree last. Fig. 2A shows the ordering of the 23 topologically different trees of 8 terminal segments. Note that the ordering is only used for presentation purposes and does in no way affect the results.

### Quantifying burst firing

Bursting is defined as the occurrence of two or more successive spikes with short interspike intervals followed by a relatively long interspike interval. To quantify bursting, we used the burst measure developed in [41]. This measure is based solely on spike times and detects the correlated occurrence of one or more short (intraburst) interspike intervals followed by a long (interburst) interspike interval. It quantifies the extent of bursting in the whole spike train; it does not try to identify individual bursts, as some other approaches do [42]. The burst measure is based on the following notion (see also Text S1). If a spike train consists of spikes with independent successive interspike intervals, the variance of the sum of two successive interspike intervals [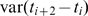, where 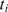 is the time of the *i*th spike in the spike train] is twice the variance of the single interspike intervals [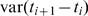]. If bursting occurs, successive intervals are no longer independent, and this relation is violated. Thus, the difference between the two variances is a measure for bursting. If we divide this difference by the squared average interspike interval, we obtain a normalized burst measure (called *B*) that is sensitive only to the relative sizes of the interspike intervals and not to the average interval size:
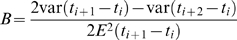
(1)where 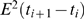 means 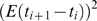, and 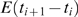 stands for taking the expectation or average value of the interspike intervals between two successive spikes in the spike train. We used eqn (1) to quantify the extent of bursting in a spike train, thus taking into account all interspike intervals 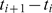 and 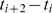 to calculate the average interspike intervals and their variances. If a spike train consists of spikes with independent successive interspike intervals (i.e., no bursting),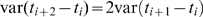, and 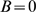. Although *B* is a continuous measure assessing the degree of bursting and not classifying spike trains as either bursting or non-bursting, we will for practical purposes consider spike trains with a value of *B* below 0.15 as non-bursting (see also Fig. S8).

In Text S1, we derive that, for a periodic spike train with two-spike bursts, the expected value of *B* is
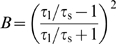
(2)where 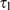 is the average interspike interval between two consecutive bursts and 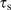 is the average interspike interval within a burst. If 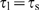, there is no bursting, and 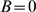. The higher the ratio 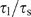 of inter- to intraburst interspike intervals, the stronger the bursting and the higher the value of 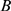. In the limiting case if 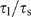 goes to infinity, *B* goes to 1. The burst measure in eqn (1) is a general measure for detecting bursting in a spike train, and in Text S2 we show that it is equally valid for spike trains containing bursts that consist of more than two spikes.

### Input conductance and mean electrotonic path length

The input conductance of a pyramidal cell with a given dendritic morphology was determined by applying a static, subthreshold current injection at the soma. The ratio of the magnitude of the injected current to the resulting change in membrane potential at the soma is defined as the input conductance of the cell [43]. The input conductance is the reverse of the input resistance.

To quantify the electrotonic extent of a dendritic tree, we introduce a new measure called mean electrotonic path length (MEP). For a given terminal segment (see Fig. 2B), the electrotonic path length is the length (normalized to the electrotonic length constant) of the path from the tip of the segment to the soma. This electrotonic path length is determined for each terminal segment, and the sum of all electrotonic path lengths is divided by the total number of terminal segments to obtain the MEP of the dendritic tree. More precisely, to obtain the MEP of a dendritic tree, we first normalize the length 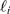 of each terminal, intermediate or root segment *i* (see Fig. 2B) with respect to its electrotonic length constants 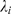, yielding a dimensionless electrotonic length 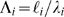 [44], in which 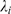 is defined as [43]

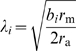
(3)where 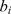 is the radius of dendritic segment *i*, and 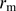 and 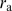 are constants denoting the specific membrane resistance and the intracellular resistivity, respectively. The MEP of a dendritic tree with 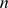 terminal segments is then given by
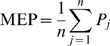
(4)where 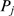 is the sum of the electrotonic lengths 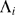 of all the dendritic segments in the path from the tip of terminal segment 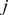 to the soma.

The analysis and model code for this paper including a tool for NEURON parameter scanning is available from ModelDB at http://senselab.med.yale.edu/modeldb via accession number 114359.

## Results

Employing a standard model of a bursting pyramidal cell [18], we investigated how dendritic morphology influence burst firing by varying either the size or the topology of the apical dendrite. The neurons were activated either at the soma with a fixed current injection or along the dendritic tree with random synaptic input. To facilitate a more comprehensive analysis, we also examined a set of morphologically simplified cells with systematic differences in dendritic topology.

### Pyramidal cell

#### Dendritic size

**Figure 3:**
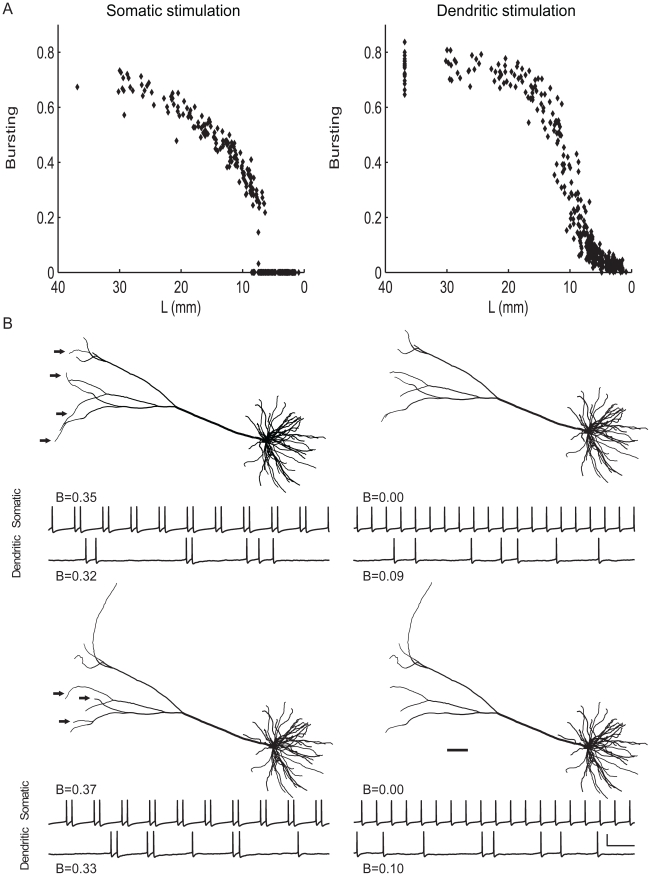
Both with somatic and with dendritic stimulation, pyramidal cell burst firing decreases as the apical dendrite becomes shorter. **A**, The degree of bursting, as measured by the burst measure *B*, against the total length of the apical dendrite. Starting with the cell in Fig. 1A, we reduced the total length by successively pruning terminal segments off the apical dendrite. **B**, Examples from the experiment in A showing that the removal of only a few small terminal segments from the apical dendritic tree can change the firing state of the cell. Morphology of pruned pyramidal cells, and voltage traces for both somatic and dendritic stimulation. *Left*, Bursting cells (*Top*, 9772 µm; *Bottom*, 8925 µm). *Right*, Non-bursting cells (*Top*, 8184 µm; *Bottom*, 6927 µm). Scale bar: 100 ms, 50 mV. Scale bar (anatomy): 100 µm. Arrows in the bursting cells indicate the branches that are shorter or absent in the non-bursting cells.

To investigate how pyramidal cell size influences burst firing, we changed the total length of the apical dendrite according to two methods. In the first method, we successively pruned terminal branches of the apical dendrite. Regression of pyramidal apical dendrities has been observed in response to, for example, chronic stress [27]–[29] (see further Discussion). The results show that both with somatic and with dendritic stimulation, the degree of bursting decreases as the dendritic tree becomes shorter (Fig. 3A; see also Fig. S8). Reducing the size of the apical dendrite ultimately transforms the bursting pyramidal cell into a tonically firing cell. The removal of only a few small terminal segments can thereby be enough to completely change the firing state of the cell (Fig. 3B). Because of the random activation of synapses, the changes in the degree of bursting are more gradual with dendritic than with somatic stimulation. For the rest, the results obtained under both stimulation regimes are similar.

**Figure 4 pcbi-1000781-g004:**
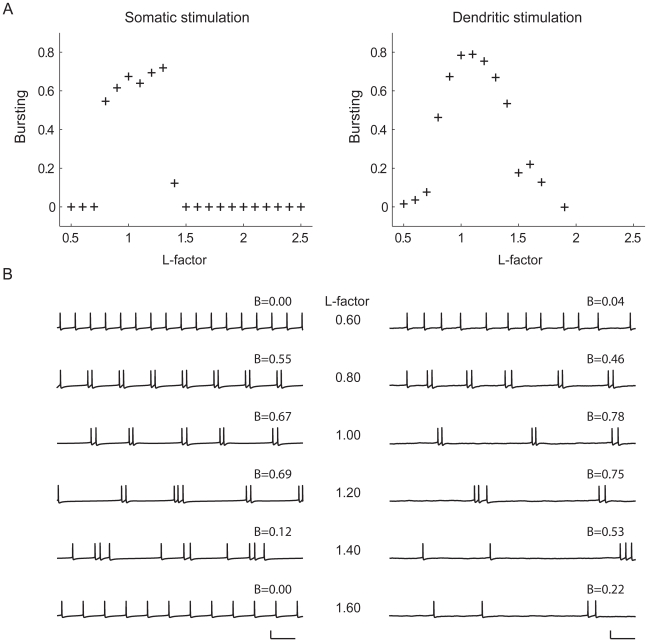
Both with somatic and with dendritic stimulation, pyramidal cell burst firing disappears when the apical dendrite becomes either too large or too small. Using the cell from Fig. 1A, we varied the size of the apical dendrite by scaling the entire apical dendrite, thus keeping the dendritic arborization intact. **A**, The degree of bursting against the factor by which the length of all the apical dendritic segments was multiplied. **B**, Voltage traces obtained for different sizes of the apical dendrite. *Left*, somatic stimulation. *Right*, dendritic stimulation. Scale bars: 100 ms, 50 mV.

In the second method, we kept the dendritic arborization intact and varied the size of the apical dendritic tree by multiplying the lengths of all its segments by the same factor. Thus, the entire apical dendritic tree was compressed or expanded. Both with somatic and with dendritic stimulation, and in line with the previous results, burst firing disappears as the total dendritic length is decreased (Fig. 4A). Interestingly, the pyramidal cell also does not exhibit burst firing when the apical dendrite becomes too large. Only when the length of the apical dendrite remains within a certain range are bursts generated. Fig 4B shows the firing patterns of the pyramidal cell at increasing lengths of its apical dendrite. When the dendrite is small, the cell does not produce bursts. At a higher dendritic length, the tonic pattern is converted into a burst pattern. Further enlarging the apical dendrite changes the frequency and fine structure of the bursts, until the cell reverts to a non-bursting state.

With somatic stimulation, the transitions from tonic firing to bursting and from bursting to tonic firing occur quite abruptly as dendritic length is modified. As with pruning, this implies that a small change in dendritic length can have a large effect on the firing state of the cell. Because of the stochastic nature of the activation of synapses, the shifts in firing state are more gradual with dendritic than with somatic stimulation, especially from bursting to tonic firing. However, the results obtained under both stimulation regimes are very much comparable, with even the onset and cessation of bursting taking place at approximately the same dendritic sizes.

#### Dendritic topology

**Figure 5:**
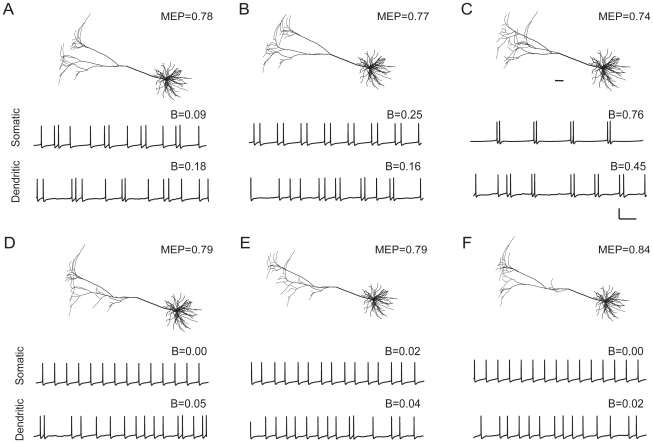
Both with somatic and with dendritic stimulation, dendritic topology affects pyramidal cell burst firing. Using the cell from Fig. 1A, we varied the topology of the apical dendritic tree by swapping branches within the tree. Thus, all the pyramidal cells shown have exactly the same total dendritic length and dendritic surface area and differ only in the topology of their apical dendrite (basal dendrites are the same). Voltage traces for three bursting cells (A–C), and three non-bursting cells (D–F). Scale bar: 100 ms, 50 mV. Scale bar (anatomy): 100 µm. MEP values indicate the mean electrotonic path length of the apical dendritic tree.

To examine whether dendritic branching structure, or topology, could influence burst firing, we varied the topology of the apical dendritic tree by swapping branches within the tree. Thus all the dendritic trees that were generated in this way have exactly the same total length and other metrical properties such as total surface area and differ only in the way their branches are connected. In this set of pyramidal cells, we find cells that produce firing patterns ranging from tonic firing to strongly bursting (Fig. 5). Just remodeling the topology of the apical dendrite can completely change the firing state of the cell and turn a bursting cell into tonically firing cell or vice versa. Interestingly, dendritic topology not only affects whether a cell exhibits bursting or not (Fig. 5A–C versus Fig. 5D–F), but also influences the fine structure or degree of bursting. The cell displayed in Fig. 5A generates (with somatic stimulation) two-spike bursts alternating with single spikes. The cells in Figs. 5B and 5C both produce a pattern of two-spike bursts, but the relative sizes of the interspike intervals between and within bursts are different. Although dendritic stimulation introduces irregularities in firing pattern because of the stochastic nature of the activation of synapses, we find that somatic and dendritic stimulation yield very much comparable results. This is rather surprising because the dynamics of action potential propagation into the dendrites and the return of current back into the soma, which underlies the ping-pong mechanism for burst generation, may completely alter with widespread synaptic stimulation of the dendritic tree owing to changes in the activation states of dendritic ion channels [45], [46].

### Morphologically simplified cells

To facilitate a better understanding of our findings obtained with the pyramidal cell model and to analyse more precisely the role of dendritic morphology in shaping burst firing, we also investigated a set of 23 morphologically simplified neurons consisting of all the topologically different trees with 8 terminal segments. Because the cells have relatively few terminal segments, the impact of dendritic topology on burst firing can be studied in a systematic way.

#### Somatic stimulation

**Figure 6:**
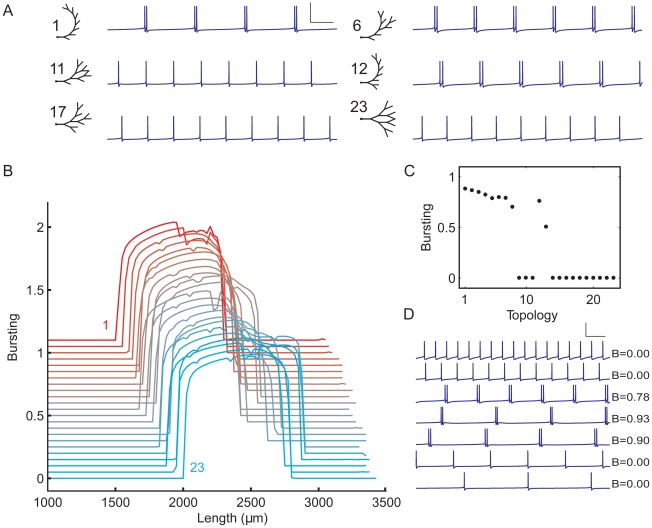
Dendritic topology influences burst firing, as demonstrated in the set of morphologically simplified neurons (Fig. 2) using somatic stimulation. The segment diameters in the trees obey Rall's power law. **A**, Examples from the set of 23 tree topologies together with corresponding voltage traces. Each tree has a total dendritic length of 1750 µm. Scale bar: 100 ms, 100 mV. **B**, The range of tree sizes where burst firing occurs is modulated by dendritic topology. For all the 23 tree topologies, the degree of bursting is shown as a function of the total length of the dendritic tree, plotted with an offset (for all horizontal lines *B* = 0) for clarity. The topologies are ordered as in Fig. 2, with the top line representing topology 1, and the bottom line topology 23. The total dendritic length of a given tree topology was varied by multiplying the lengths of all its segments by the same factor. **C**, Bursting as a function of topology at a total dendritic length of 1750 µm. **D**, For topology 1, voltage traces at different total dendritic lengths, together with *B* values indicating the degree of bursting. From top to bottom: 1000, 1300, 1600, 1900, 2200, 2500, and 2800 µm. Scale bar: 100 ms, 100 mV.

To show how both dendritic length and dendritic topology affect bursting, we plotted in Fig. 6B for all the 23 tree topologies the degree of bursting, under somatic stimulation, as a function of the total length of the dendritic tree. The total length of a given tree topology was varied by changing the lengths of all the segments in the tree by the same amount. As in the full pyramidal cell model, bursting occurs only for a certain range of tree sizes. Interestingly, this range depends on the topology of the dendritic tree: trees with an asymmetric branching structure start bursting at a lower total dendritic length than trees with a symmetric branching structure, and also stop bursting at a lower total dendritic length. The transitions from tonic firing to bursting and from bursting to tonic firing are not gradual but occur quite abruptly (Fig 6B) as the dendritic tree is enlarged. Fig 6D shows the firing patterns in a fully asymmetrical tree (topology 1) at different tree sizes. With increasing size, the firing pattern goes from high-frequency tonic firing to bursting to low-frequency tonic firing.

Because different tree topologies start and stop bursting at different total lengths, a change in the topology of the tree, while keeping the size of the tree the same (i.e., a change along a vertical line through Fig. 6B), can already shift the firing pattern from bursting to non-bursting or vice versa. For example, with somatic stimulation and at a total dendritic length of 1750 µm, the firing pattern shifts from bursting to tonic firing as the topological structure is changed from asymmetric to symmetric (Fig. 6C; see also Fig. 6A).

#### Dendritic stimulation

**Figure 7:**
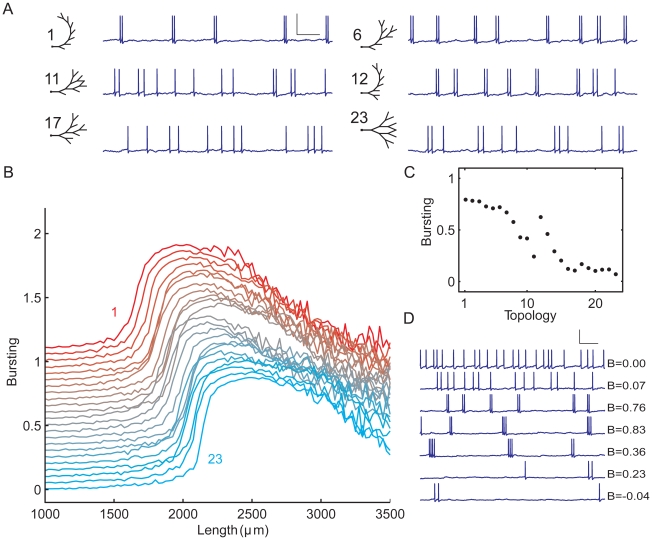
Dendritic topology influences burst firing, as demonstrated in the set of morphologically simplified neurons using dendritic stimulation. The segment diameters in the trees obey Rall's power law. **A**, Examples from the set of 23 tree topologies together with corresponding voltage traces. Each tree has a total dendritic length of 1900 µm. Scale bar: 100 ms, 100 mV. **B**, As with somatic stimulation, the range of tree sizes where burst firing occurs is affected by dendritic topology. For all the 23 tree topologies, the degree of bursting is shown as a function of the total length of the dendritic tree, plotted with an offset for clarity. The topologies are ordered as in Fig. 2, with the top line representing topology 1, and the bottom line topology 23. The total dendritic length of a given tree topology was varied by multiplying the lengths of all its segments by the same factor. **C**, Bursting as a function of topology at a total dendritic length of 1900 µm. **D**, For topology 1, voltage traces at different total dendritic lengths, together with *B* values indicating the degree of bursting. From top to bottom: 1000, 1400, 1800, 2200, 2600, 3000, and 3400 µm. Scale bar: 100 ms, 100 mV.

As with somatic stimulation, with dendritic stimulation there is also only a range of tree sizes where bursting occurs and this range depends on topological structure, with asymmetric trees starting bursting at lower total lengths than symmetric trees (Fig. 7B). For each tree topology, the onset of bursting takes place at approximately the same total length as with somatic stimulation. As in the full pyramidal cell model, the firing patterns are more irregular and the transitions in firing state more gradual than with somatic stimulation, especially from bursting to tonic firing. For a given tree topology, the firing pattern goes from high-frequency tonic firing to bursting to low-frequency tonic firing as dendritic size is increased (Fig 7D). Interestingly, Fig 7D shows that dendritic size can also affect the number of spikes per burst. When the dendritic tree is large enough that the cell is capable of generating bursts, it does so with two-spike bursts. Further increasing the dendritic length then yields a firing pattern with up to 4 spikes per burst. As with somatic stimulation, a change in only the topology of the dendritic tree can markedly change the degree of bursting (Fig. 7A and C).

#### Dendritic trees with uniform segment diameters

**Figure 8:**
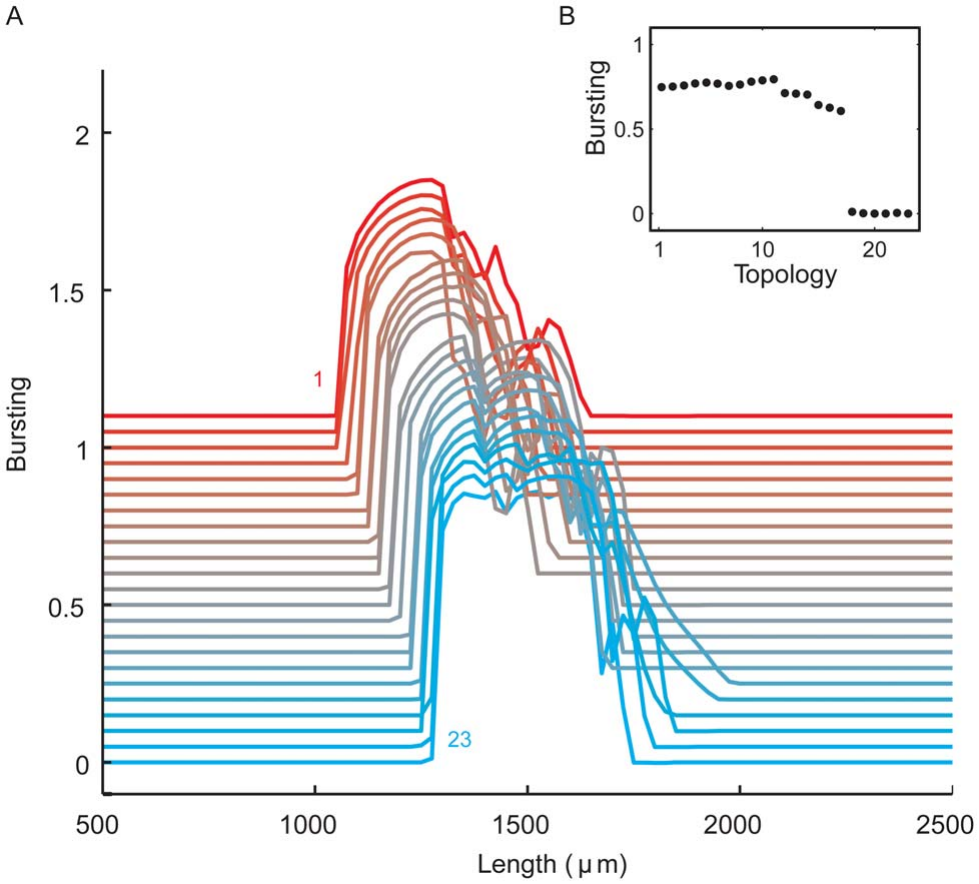
Dendritic topology influences burst firing also if all segment diameters are uniform throughout the whole tree. Thus, all the 23 tree topologies have the same total dendritic surface area as well the same total dendritic length. **A**, For all the 23 tree topologies, the degree of bursting is shown as a function of the total length of the dendritic tree, plotted with an offset for clarity, and using somatic stimulation. The topologies are ordered as in Fig. 2, with the top line representing topology 1, and the bottom line topology 23. The total dendritic length of a given tree topology was varied by multiplying the lengths of all its segments by the same factor. **B**, Bursting as a function of topology at a total dendritic length of 1250 µm.

To analyse further how dendritic morphology controls burst firing, we also studied the set of 23 tree topologies with all dendritic segments of a tree having exactly the same diameter. If dendritic diameters obey Rall's power law, as they do in Figs. 6 and 7, asymmetrical topologies have a higher total surface area than symmetrical topologies (see Methods). Therefore, we might hypothesize that dendritic surface area, which affects the input conductance at the soma, underlies the influence of dendritic topology on burst firing. Fig. 8, in which all segment diameters are uniform and all tree topologies consequently have exactly the same total surface area, shows that this is not the case. We observe the same impact of dendritic topology (and size) on burst firing as in the trees obeying Rall's law, with again asymmetric trees starting bursting at lower total lengths than symmetric trees (Fig. 8A). Note that this is also in agreement with our findings in the pyramidal cell with full morphological complexity, in which all the permutated branching structures have exactly the same total surface area (and length) and nevertheless exhibit different firing patterns.

#### Input conductance and mean electrotonic path length

**Figure 9:**
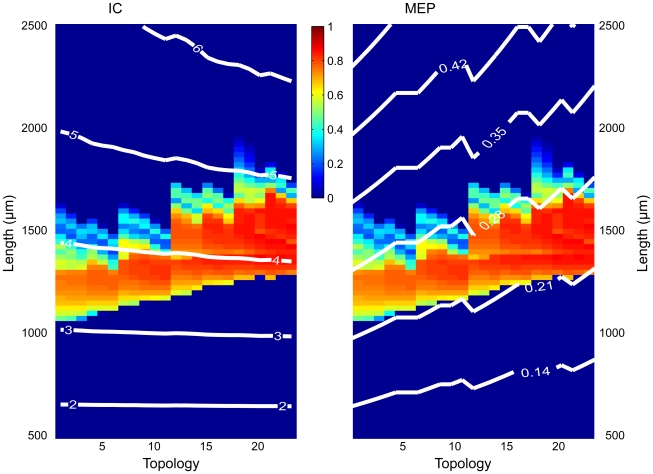
Mean electrotonic path length, but not input conductance, correlates with the region of burst firing for trees with uniform segment diameters and under somatic stimulation. Except for the white contour lines, the left and right panels are identical and show the degree of burst firing (color coded) as a function of both dendritic topology and total dendritic length. In the left panel, contour lines of equal input conductance (IC, in µS) are superimposed. These contour lines show all the combinations of dendritic length and dendritic topology that result in a given input conductance. Similarly, in the right panel, contour lines of equal mean electrotonic path length (MEP, in units of the electrotonic length constant) are superimposed. These contour lines show all the combinations of dendritic length and dendritic topology that result in a given MEP value. The onset and cessation of bursting are strongly associated with mean electrotonic path length, but not with input conductance.

Although in Fig. 8 the total dendritic surface area, for a given dendritic length, is equal for all tree topologies, the tree topologies still differ in input conductance, because of the connectivity structure of the dendritic segments. In asymmetrical trees, the dendritic segments are connected more in series than they are in symmetrical trees, so that the input conductance of asymmetrical trees is lower than that of symmetrical trees [20]. Consequently, differences in input conductance might still be responsible for the effect of dendritic topology on burst firing. In Fig. 9, we plot, for trees with uniform segment diameters, the degree of burst firing (color coded) for different dendritic topologies and tree sizes, together with contour lines of equal input conductance. This reveals that neither the onset nor the cessation of bursting is associated with a particular input conductance. Thus, differences in input conductance cannot account for the influence of dendritic morphology on burst firing.

What instead appears to be a critical factor is the electrotonic extent of the dendritic tree, as measured by the mean electrotonic path length. The mean electrotonic path length of a dendritic tree is the sum of all electrotonic dendritic path lengths measured from the tip of a terminal segment to the soma divided by the total number of terminal segments (see Methods). For trees with uniform segment diameters, Fig. 9 depicts the degree of burst firing for different topologies and tree sizes, together with contour lines of equal mean electrotonic path length. This reveals that both the onset and the cessation of bursting are strongly related to mean electrotonic path length, with burst firing occurring only within a certain range of values.

**Figure 10:**
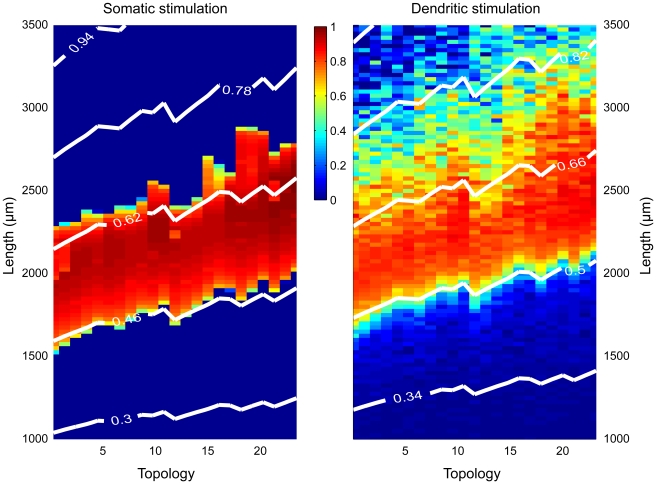
Mean electrotonic path length correlates with the region of burst firing for trees whose segment diameters obey Rall's power law. The degree of burst firing (color coded) is shown as a function of both dendritic topology and total dendritic length, under somatic stimulation (*Left*) and dendritic stimulation (*Right*). The superimposed white lines are contour lines of equal mean electrotonic path length (MEP, in units of the electrotonic length constant), showing all the combinations of dendritic length and dendritic topology that result in a given MEP value. Especially the onset of burst firing is strongly associated with mean electrotonic path length.

We now return to the set of tree topologies in which the segment diameters obey Rall's power law, to see whether mean electrotonic path length is also a determining factor for dendritic trees with a more realistic distribution of diameter sizes. For both somatic and dendritic stimulation, Fig. 10 shows that there is indeed a remarkably strong correlation between onset of bursting and mean electrotonic path length. Burst firing occurs when the mean electrotonic path length is higher than a certain critical value. At the same total dendritic length, asymmetrical trees have a higher mean electrotonic path length than symmetrical trees, and consequently reach this critical value earlier than symmetrical trees as dendritic length is increased. The cessation of bursting, especially with somatic stimulation, is also clearly related to mean electrotonic path length, albeit less precise than for the onset of bursting. Thus, the combined effect of dendritic size and dendritic topology on burst firing can be captured to a large extent by the mean electrotonic path length of the dendritic tree.

#### Importance of electrotonic distance and dendritic topology for burst firing

**Figure 11:**
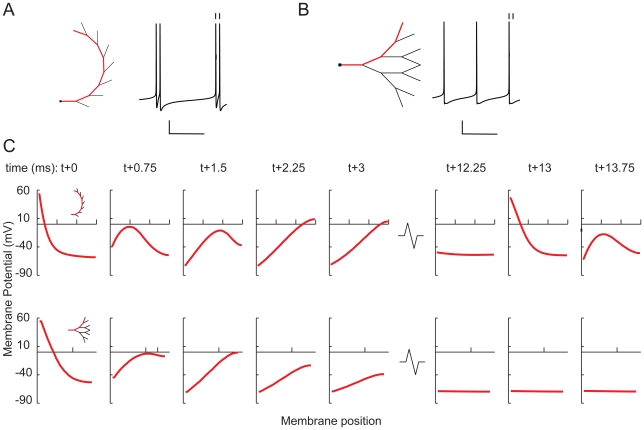
The importance of electrotonic distance for burst firing and the impact of dendritic topology illustrated with a fully asymmetrical and a fully symmetrical tree. **A**, **B**, At this dendritic size, the asymmetrical tree (**A**) generates bursts, whereas the symmetrical tree (**B**) produces single spikes. The segment diameters in the trees obey Rall's power law, and both trees have the same total dendritic length (1600 µm). Scale bars: 100 ms, 20 mV. The ticks on top of the action potentials in A and B indicate the spikes that are shown at t+0 and t+13 in panel C. **C**, The membrane potential evolution over time in the asymmetrical tree (*top row*) and the symmetrical tree (*bottom row*) along the dendritic paths indicated in A and B. Time is relative to the first spike (at 0 ms), and membrane position on the x-axis runs from soma to the tip of the terminal segment. Because the distance between soma and terminal segment is smaller in the symmetrical than in the asymmetrical tree, the membrane potential evolution in the symmetrical tree has less spatial differentiation, the membrane potential reaches a lower value at the distal end, and the distal membrane potential start decreasing earlier in time, so that the return current from dendrites to soma reaches the soma at a time when the delayed-rectifier K^+^ channels are still open, preventing the generation of a second spike. See further Results.

We next provide an explanation for the importance of mean electrotonic path length for bursting, which is further supported in Fig. 11. In the burst firing mechanism of pyramidal cells, a somatically generated action potential propagates into the dendritic tree and depolarizes the dendrites, creating a potential difference between distal dendrites and soma. This leads to a return current from dendrites to soma, which, if strong enough, produces another somatic spike (‘ping-pong’ mechanism) [12], [13] (see also Introduction). The arrival of the backpropagating action potential in the dendritic tips marks the onset of the return current (see also Fig. 11). The dendritic tips are, as it were, the reflection points of the backpropagating action potential, where the current starts to move back to the soma. If these return currents reach the soma when the delayed-rectifier K^+^ conductance is still high, it will be difficult for the soma to depolarize and produce a spike. Since the propagation velocity of voltages and currents is proportional to the electrotonic length constant [44], the mean electronic path length is a measure for the average time it takes for a backpropagating action potential to travel to the dendritic tips (and for the return current to move to the soma). Thus, if the mean electronic path length is too small, the return current will arrive too early at the soma, when the delayed-rectifier K^+^ conductance is still high, so that it cannot produce another spike—that is, no bursting.

Furthermore, if the electrotonic distance between soma and distal dendrites is too small, the large conductive coupling will lead to currents that quickly annul membrane potential differences between soma and distal dendrites. This prohibits a strong and long-lasting differentiation in membrane potential between soma and distal dendrites, which is the generator of the return current that lies at the heart of the ping-pong mechanism of bursting. In the limiting case, with very small electrotonic distance, soma and dendrites can be considered as a single compartment with a uniform potential.

If the electrotonic distance between soma and distal dendrites is too large, bursting will also not occur. Note that even in the absence of a return current, the cell will generate a next spike as a result of the external (somatic or dendritic) stimulation. So, what the return current in fact does when it causes bursting is to advance the timing of the next spike. If the electronic distance is too large, the return current will arrive too late—that is, not before the external stimulation has already caused the cell to spike. Furthermore, if the electrotonic distance is too large, the potential gradient between distal dendrites and soma will become too shallow for a strong return current. In summary, for the soma and distal dendrites to engage in a ping-pong interaction, the electrotonic distance between the two should be neither too small nor too large.

Thus, when the mean electrotonic path length becomes too small or too large as the total size of the dendritic tree is varied, as in Figs. 9 and 10, bursting will not occur. Importantly, the mean electrotonic path length depends also on the topology of the dendritic tree, which accounts for the influence of dendritic topology on burst firing. In asymmetrical trees, the terminal segments are on average further away from the soma than in symmetrical trees. Consequently, at the same dendritic size, asymmetrical trees have a higher mean electrotonic path length—as well as ‘normal’ mean path length—than symmetrical trees. As a result, asymmetrical trees reach the critical values of mean electrotonic path length from where bursting starts, and from where it stops, at lower dendritic sizes than symmetrical trees (Figs. 9 and 10).

To illustrate the importance of electrotonic distance for bursting and the impact of topological structure, we show in Fig. 11 the potential evolution over time along the dendrites of a fully asymmetrical and a fully symmetrical tree. Both trees have the same total dendritic length. At this dendritic size, the asymmetrical tree exhibits burst firing and the symmetrical one does not. The spatiotemporal dynamics of membrane potential along the dendrite of the symmetrical tree shows two main differences compared with that of the asymmetrical tree. First, the potential evolution has less spatial differentiation. In the asymmetrical tree, the electrotonic (and normal) distance between soma and distal dendrites is large enough for a full propagating wave to develop (between *t*+0.75 and *t*+1.5), whereas in the symmetrical tree it is not. The membrane potential at the distal end also reaches a lower value than in the asymmetrical tree. Second, although the membrane potential at the distal end of the symmetrical tree decreases between *t*+1.5 and *t*+3, this does not result in an increase in the membrane potential at the soma. In the symmetrical tree, the distal membrane potential start decreasing earlier in time than in the asymmetrical tree, so that the return current from the dendrites will arrive at the soma when the delayed-rectifier K^+^ channels are still open, hampering somatic depolarization. Consequently, the membrane potential is not raised enough to trigger another spike, whereas in the asymmetrical tree it is (between *t*+12.25 and *t*+13.75).

**Figure 12:**
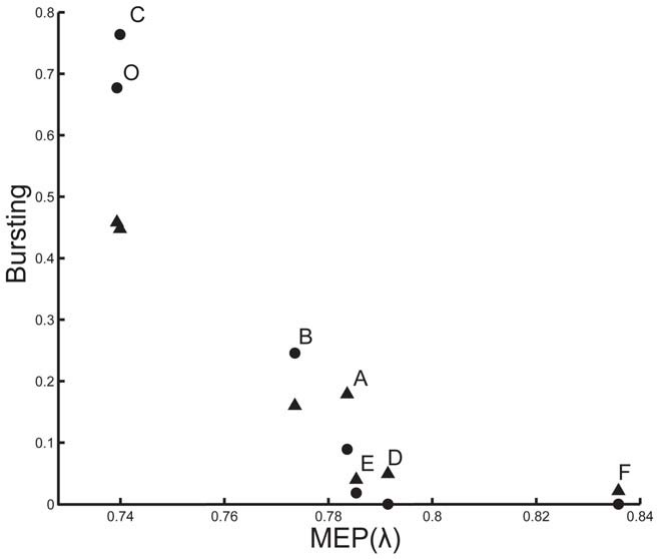
Burst firing in the pyramidal cells of Fig. 5 correlates strongly with mean electrotonic path length (MEP, in units of the electrotonic length constant), both with somatic stimulation (*circles*; *r* = −0.97) and dendritic stimulation (*triangles*; *r* **= −0.99).** (In the calculation of the correlation, we excluded the two MEP values around 0.84, thus ignoring the part of the curve that is clearly flat). The letters A–F correspond to those in Fig. 5, and O indicates the unperturbed pyramidal cell of Fig. 1.

We now return to the pyramidal cells with full morphological complexity, to see whether burst firing is also there related to mean electrotonic path length. In Fig. 12, we plot the mean electrotonic path length against the degree of bursting for the set of permutated pyramidal cells in Fig. 5. All these cells have exactly the same total dendritic length and dendritic surface area and differ only in the arrangement of their branches. Fig. 12 shows that burst firing indeed strongly correlates with mean electrotonic path length, for both somatic and dendritic stimulation.

In general, the results obtained with dendritic stimulation are comparable to those produced with somatic stimulation (Figs. 3–5, Fig. 6 vs Fig. 7, Fig. 10, Fig. 12). In the ping-pong mechanism of burst firing, the sequence and timing of events start when a somatic action potential propagates into the dendritic tree. How this action potential is generated in the first place, by current injection into the soma or as a result of summation of dendritic synaptic inputs, appears not to be crucial. The dynamics of action potential propagation and the return of current back into the soma may be modulated by synaptic stimulation of the dendritic tree, but also under dendritic stimulation the mean electrotonic path length serves as the functionally relevant measure for the time it takes for the propagation of signals between soma and dendritic tips.

## Discussion

Given the crucial role of bursts of action potentials in synaptic plasticity and neuronal signaling, it is important to determine what factors influence their generation. Using a standard compartmental model of a reconstructed pyramidal cell [18], we have investigated how size and topology of dendritic morphology affect intrinsic neuronal burst firing.

We have shown that either shortening or lengthening the apical dendrite tree beyond a certain range can transform a bursting pyramidal cell into a tonically firing cell. Remarkably, altering only the topology of the dendritic tree, whereby the total length of the tree remains unchanged, can likewise shift the firing pattern from bursting to non-bursting or vice versa. Moreover, both dendritic size and dendritic topology not only influence whether a cell is bursting or not, but also affect the number of spikes per burst and the interspike intervals between and within bursts.

The influence of dendritic morphology on burst firing is attributable to the effect dendritic length and dendritic topology have, not on input conductance, but on the spatial extent of the dendritic tree, as measured by the mean electrotonic path length between soma and distal dendrites. For the spatiotemporal dynamics of dendritic membrane potential to generate burst firing, this electrotonic distance should be neither too small nor too large. Because the degree of symmetry of the dendritic tree also determines mean electrotonic path length, with asymmetrical trees having larger mean path lengths than symmetrical trees, dendritic topology as well as dendritic size affects the occurrence of burst firing.

In Mainen and Sejnowski's [18] two-compartment model for explaining the role of dendritic morphology in shaping firing pattern, the spatial dimension of morphology was completely reduced away. Although the model is able to reproduce a wide range of firing patterns, it does not capture the essential influence of dendritic morphology on burst firing, in which, as we have shown here, the spatial extent of the dendritic tree and the resulting spatiotemporal dynamics of the dendritic membrane potential are crucially involved.

The effect of dendritic size and topology on burst firing and the correlation of burst firing region with mean electrotonic path length are robust to changes in model properties, including morphology, strength of input stimulus, ion channel densities, and keeping the number of ion channels constant as morphology is changed. The specific range of dendritic sizes that supports burst firing, as well as the impact of dendritic topology, does not strongly depend on the strength of the input stimulus, especially with somatic stimulation (Figs. S1 and S2). More importantly, the overall way in which dendritic morphology influences burst firing is independent of stimulus strength. Likewise, the impact of dendritic morphology is qualitatively insensitive to the value of the branch power used in the morphologically simplified cells: even in dendritic trees in which the segment diameters are uniform we observe the same effect of dendritic length and topology (Fig. 8).

In changing dendritic size or topology, we held the density of ion channels constant (i.e., the conductances were fixed), which implies that the total number of ion channels also changed when dendritic morphology was varied. Keeping the conductances fixed seems biologically the most appropriate choice, since removing membrane to shrink the dendritic tree (as well as adding membrane to enlarge it) will include the membrane's ion channels and is therefore not expected to affect ion channel density. But even if we hold the number of ion channels constant, by adjusting the values of the conductances as the surface area of the dendritic tree is changed when dendritic topology or total length is varied, we obtain surprisingly similar results (Fig. S3). Although the precise values of mean electrotonic path length that support burst firing are slightly different, the overall effect of dendritic size and topology on burst firing and the correlation of burst firing region with mean electrotonic path length remain the same.

Since recent studies have shown that the same firing patterns can be produced by different combinations of conductances [47], [48] and even by different combinations of conductances and morphological properties [49], it is important to ensure that our results are not specific for the particular choice of conductance values in the Mainen and Sejnowski model [18]. Provided the model supports the ping-pong mechanisms of burst firing, our main findings are indeed robust to considerable changes in ion channel densities, both under somatic and under dendritic stimulation. Although the range of tree sizes that supports burst firing may be different for different ion channel densities, Figs. S4, S5, S6, S7 show that the general impact of dendritic size and topology on burst firing, as well as the correlation of burst firing with mean electrotonic path length, is maintained for a wide range of dendritic ion channel densities. Interestingly, the value of the mean electrotonic path length where burst firing commences, going from small to large trees, is not affected by ion channel density, as opposed to the value of the mean electrotonic path length where burst firing stops. This suggests that when the dendritic tree is reduced in size so that the cell no longer exhibits burst firing, compensatory changes in ion channel conductances [49] may not be able to bring back the cell to a bursting mode. In contrast, when the dendritic tree is enlarged beyond the range where the cell bursts, compensatory changes in ion channel conductances (e.g., increased dendritic 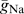) may be able to restore burst firing.

Since it has experimentally been shown that removal of the apical dendrite abolishes bursting in layer 5 pyramidal cells [50] and pathological conditions often affect the apical dendrite, but not the basal dendrites [29], we focused in this study on the morphology of the apical dendritic tree. The simulations with the morphologically simplified cells, which do not have basal dendrites, show that basal dendrites are not essential for burst firing. In the pyramidal cell and the simplified cells, burst firing is similarly affected by dendritic morphology, which again emphasizes the robustness of our findings.

Compared with other modeling studies investigating the relationship between dendritic morphology and firing pattern [18]–[21], [51], our study is unique in that it focuses on burst firing, adopts a systematic approach, investigates morphological changes within a cell type, considers not only somatic stimulation but also physiologically more appropriate dendritic stimulation, and especially examines the impact of topological structure of dendritic arborizations. Moreover, our study is not just correlative but provides insight into the mechanisms underlying the influence of morphology on firing pattern.

We stimulated the cells either by a current injection at the soma, as is done in most experimental and modeling studies [18], or by synapses distributed over the dendritic tree, which is physiologically more relevant. Importantly, we found that the influence of dendritic morphology on burst firing is essentially the same under both stimulation regimes.

Our study confirms a suggestion by Krichmar et al. [21] that dendritic branching structure might have a direct influence on neuronal firing activity. In simulation studies, they found that although dendritic size could account for much of the differences in neuronal firing behavior between CA3 pyramidal cells, it did not provide a complete explanation for the observed electrophysiological variability.

Our results are in accord with empirical observations suggesting that pyramidal cells should have reached a minimal size to be capable of burst firing. In weakly electric fish, the tendency of pyramidal cells to fire bursts is positively correlated with the size of the cell's apical dendritic tree [52]. In rat prefrontal cortex [16] and visual cortex [14], [53], the classes of pyramidal cells that exhibit burst firing have a greater total dendritic length than the other classes.

In addition, the developmental time course of bursting shows similarities with that of dendritic morphology. In rat sensorimotor cortex, the proportion of bursting pyramidal cells progressively increases from postnatal day 7 onwards, while at the same time the dendritic arborizations become more complex [54]. In pyramidal cells from rat prefrontal cortex, the total lengths of apical and basal dendrites increase dramatically between postnatal days 3 and 21, with neurons capable of burst firing appearing only from postnatal day 18 onwards [55], [56].

Direct experimental testing of the influence of dendritic morphology on burst firing could be done by physically manipulating the shape or size of the dendritic tree, e.g., by using techniques developed by Bekkers and Häusser [50]. They showed that dendrotomy of the apical dendrite indeed abolished bursting in layer 5 pyramidal cells.

Dendritic morphology can undergo significant alterations in many pathological conditions, including chronic stress [27]–[29], [57], epilepsy [26], hypoxic ischemia [58], Alzheimer [22], [23], and disorders associated with mental retardation [24], [25]. Functional consequences of these morphological changes are usually interpreted in terms of loss or formation of synaptic connections as a result of a diminished or expanded postsynaptic surface area. Our modeling results indicate that alterations in dendritic morphology can directly modify neuronal firing, irrespective of changes in total synaptic input.

Chronic stress, as well as daily administration of corticosterone, induces extensive regression of pyramidal apical dendrites in hippocampus [27], [57], [59] and prefrontal cortex [28], [29]. As a result of a decrease in the number and length of terminal branches, the total apical dendritic length can reduce by as much as 32% [29], while basal dendrites are not affected. Similarly large alterations have been observed in response to mild, short-term stress [60]. Our results predict that stress and the accompanying reduction in apical dendritic length could turn a bursting neuron into a non-bursting one. Indeed, Okuhara and Beck [61] found that two weeks of high corticosterone treatment caused a decrease in the relative number of intrinsically bursting CA3 pyramidal cells. Since burst firing of CA3 pyramidal cells is critically involved in LTP [62], this could have profound functional consequences for hippocampal information processing [63].

With regard to epilepsy, a significant decrease in total dendritic length and number of branches has been found in pyramidal cells following neocortical kindling [26]. In line with our results, Valentine et al. [64] reported that activity of single cells recorded from the primary auditory cortex of kindled cats showed a reduction in the amount of burst firing and a decrease in the number of spikes per burst.

In Alzheimer's disease, various aberrations in dendritic morphology have been observed— including a reduction in total dendritic length and number of dendritic branches [22], [23] and alterations in the pattern of dendritic arborization [65]—which may contribute to the abnormal neurophysiological properties of Alzheimer pyramidal cells [66]. The anomalies in morphology could influence the cells' ability to produce burst, and, because of the role of burst firing in LTP and LTD [8], [9], ultimately affect cognition. In disorders related with mental retardation, the observed alterations in dendritic length and pattern of dendritic branching [24], e.g., changes in the degree of symmetry of the apical dendrite [67], may likewise be hypothesized to contribute to impaired cognition. Importantly, our results indicate that dendritic sprouting—which too has been observed in Alzheimer [68], [69]—may also be able to change neuronal burst firing.

Since firing patterns characteristic of different classes of neurons may in part be determined by total dendritic length, we expect on the basis of our results that a neuron may try to keep its dendritic size within a restricted range in order to maintain functional performance. Indeed, Samsonovich and Ascoli [70] have shown that total dendritic size appears to be under intrinsic homeostatic control. Statistically analyzing a large collection of pyramidal cells from hippocampus and prefrontal cortex, they found that, for a given morphological class and anatomical location, fluctuations in dendritic size in one part of a cell tend to be counterbalanced by changes in other parts of the same cell, so that the total dendritic size of each cell is conserved.

We predict that dendritic topology may similarly be protected from large variations. In fact, there could be a trade-off between dendritic size and dendritic topology. In a set of bursting pyramidal cells, we expect that apical dendritic trees with a lower degree of symmetry are shorter in terms of total dendritic length or have thicker dendrites to reduce electrotonic length than those with a higher degree of symmetry.

Although changes in dendrite morphology of pyramidal cells have been observed in response to environmental enrichment [71] and learning [72], recent long-term in vivo imaging studies have demonstrated remarkable stability of dendrites in adult animals [73], [74]. Like the homeostatic control of dendritic size, this stability may point to the functional relevance of dendritic topology.

An intriguing possibility is that firing pattern and dendritic morphology could mutually tune each other during development, as a result of a reciprocal influence between dendritic growth and neuronal activity. Dendritic morphology affects firing pattern, and neuronal activity in turn is known to modulate dendritic growth and branching [75], with, interestingly, firing frequency and firing pattern having distinct effects on outgrowth [76].

As our study underscores, differences in neuronal firing properties may not necessarily reflect differences in ion channel composition. In some cases, variability in dendritic morphology may even have a relatively bigger effect on firing pattern than variability in membrane conductances [49], [77]. Our results show that alterations in either the size or the topology of dendritic arborizations, as have been observed in many pathological conditions, could have a marked impact on pyramidal cell burst firing and, because of the critical role of bursting in neuronal signaling and synaptic plasticity, ultimately affect cognition.

## Supporting Information

Figure S1 The region of burst firing is relatively insensitive to the strength of somatic stimulation. For three different tree topologies of the morphologically simplified cells (see Fig. 2; segment diameters according to Rall's power law), the degree of burst firing (color coded) is shown as a function of total dendritic length and relative stimulation strength (where 1 is our standard somatic current injection; see Methods). A doubling of the stimulation strength causes only a 5–10% shift in the position of the burst region. The weaker bursting for stronger stimuli is mainly the result of smaller interburst intervals.
(0.14 MB PDF)

Figure S2 The region of burst firing is relatively insensitive to the strength of dendritic stimulation. For three different tree topologies of the morphologically simplified cells (see Fig. 2; segment diameters according to Rall's power law), the degree of burst firing (color coded) is shown as a function of total dendritic length and relative stimulation strength (where 1 is our standard synaptic peak conductance; see Methods). The weaker bursting for stronger stimuli is mainly the result of smaller interburst intervals.
(0.16 MB PDF)

Figure S3 The mean electrotonic path length correlates with the region of burst firing also when the number of ion channels is kept constant as the topology or total length of the tree is changed. To implement a constant number of ion channels, we decreased (increased) the ion channel densities (i.e., maximal conductances, expressed in pS µm∧−2) as the total surface area of the dendritic tree increased (decreased). The total dendritic surface area of the fully symmetrical tree (topology 23) at dendritic length 2500 µm was thereby taken as reference. Thus, g_x new = g_x * (surface area of the symmetrical tree at 2500 µm)/(surface area of the tree under consideration), where g_x is the standard maximal conductance as given in Methods and the index x indicates channel type. As in Fig. 10, the segment diameters of the trees obey Rall's power law. (Recall that for Rall trees, a change in tree topology also results in a different total dendritic surface area; see Methods.) The degree of burst firing (color coded) is shown for different dendritic topologies and tree sizes, together with contour lines of equal mean electrotonic path length (in units of the electrotonic length constant).
(0.17 MB PDF)

Figure S4 The influence of dendritic size and topology on burst firing and the importance of mean electrotonic path length are robust to changes in ion channel densities. For a wide range of dendritic ion channel densities, the mean electrotonic path length correlates with the region of burst firing. The maximal conductance of Na is 90% of the standard value (see Methods). The maximal conductances of Km and KCa are varied. The factors f multiply the standard values of the maximal conductances. The segment diameters of the trees obey Rall's power law. The cells are stimulated by somatic stimulation. Each sub-panel, as in Figs. 9 and 10, shows the degree of burst firing (color coded) as a function of dendritic size and dendritic topology, together with contour lines of equal mean electrotonic path length (in units of the electrotonic length constant).
(0.06 MB PDF)

Figure S5 The influence of dendritic size and topology on burst firing and the importance of mean electrotonic path length are robust to changes in ion channel densities. For a wide range of dendritic ion channel densities, the mean electrotonic path length correlates with the region of burst firing. The maximal conductance of Na is 110% of the standard value (see Methods). The maximal conductances of Km and KCa are varied. The factors f multiply the standard values of the maximal conductances. The segment diameters of the trees obey Rall's power law. The cells are stimulated by somatic stimulation. Each sub-panel, as in Figs. 9 and 10, shows the degree of burst firing (color coded) as a function of dendritic size and dendritic topology, together with contour lines of equal mean electrotonic path length (in units of the electrotonic length constant).
(0.12 MB PDF)

Figure S6 The influence of dendritic size and topology on burst firing and the importance of mean electrotonic path length are robust to changes in ion channel densities. For a wide range of dendritic ion channel densities, the mean electrotonic path length correlates with the region of burst firing. The maximal conductance of Na is 90% of the standard value (see Methods). The maximal conductances of Km and KCa are varied. The factors f multiply the standard values of the maximal conductances. The segment diameters of the trees obey Rall's power law. The cells are stimulated by dendritic stimulation. Each sub-panel, as in Figs. 9 and 10, shows the degree of burst firing (color coded) as a function of dendritic size and dendritic topology, together with contour lines of equal mean electrotonic path length (in units of the electrotonic length constant).
(0.12 MB PDF)

Figure S7 The influence of dendritic size and topology on burst firing and the importance of mean electrotonic path length are robust to changes in ion channel densities. For a wide range of dendritic ion channel densities, the mean electrotonic path length correlates with the region of burst firing. The maximal conductance of Na is 110% of the standard value (see Methods). The maximal conductances of Km and KCa are varied. The factors f multiply the standard values of the maximal conductances. The segment diameters of the trees obey Rall's power law. The cells are stimulated by dendritic stimulation. Each sub-panel, as in Figs. 9 and 10, shows the degree of burst firing (color coded) as a function of dendritic size and dendritic topology, together with contour lines of equal mean electrotonic path length (in units of the electrotonic length constant). Comparison of Figs. S4, S5, S6, S7 shows that although the range of dendritic sizes that exhibits burst firing may be different for different dendritic ion channel densities (with a higher density of Na channels, the dendritic length range that shows burst firing is larger; e.g., compare Figs. S4 and S5), the overall effect of dendritic size and topology on burst firing and the correlation of the region of burst firing with mean electrotonic path length is the same in all cases. Note that the value of the mean electrotonic path length where burst firing commences is the same for different combinations of ion channels densities, both under somatic stimulation (MEP = 0.46) and under dendritic stimulation (MEP = 0.50).
(0.12 MB PDF)

Figure S8 Interspike-interval (ISI) distributions, together with burst measure values (B), in the experiment in which the total length of the pyramidal cell was gradually reduced by pruning the apical dendrite (see Fig. 3, dendritic stimulation). For every step in a single pruning sequence, the ISI distribution (bin size = 25 ms) and B value are shown. The top left graph is of the intact pyramidal cell, and every step going from left to right signifies a round of pruning (see Methods). In this experiment, B = 0.12 is already accompanied by a weakly bimodal ISI distribution, while B values larger than 0.25 are associated with marked bimodality.
(0.15 MB PDF)

Text S1 The burst measure and the derivation of its expected value for a periodic spike train with two-spike bursts.
(0.18 MB PDF)

Text S2 Burst firing described as a semi-Markov process and generalization of the burst measure to n-spike bursts
(0.13 MB PDF)
