# Supplementary figures and images for: Impact of Dendritic Size and Dendritic Topology on Burst Firing in Pyramidal Cells
Source: PLoS Comput Biol. 2010 May 13;6(5):e1000781. doi: 10.1371/journal.pcbi.1000781 (PMC2869305; doi:10.1371/journal.pcbi.1000781)

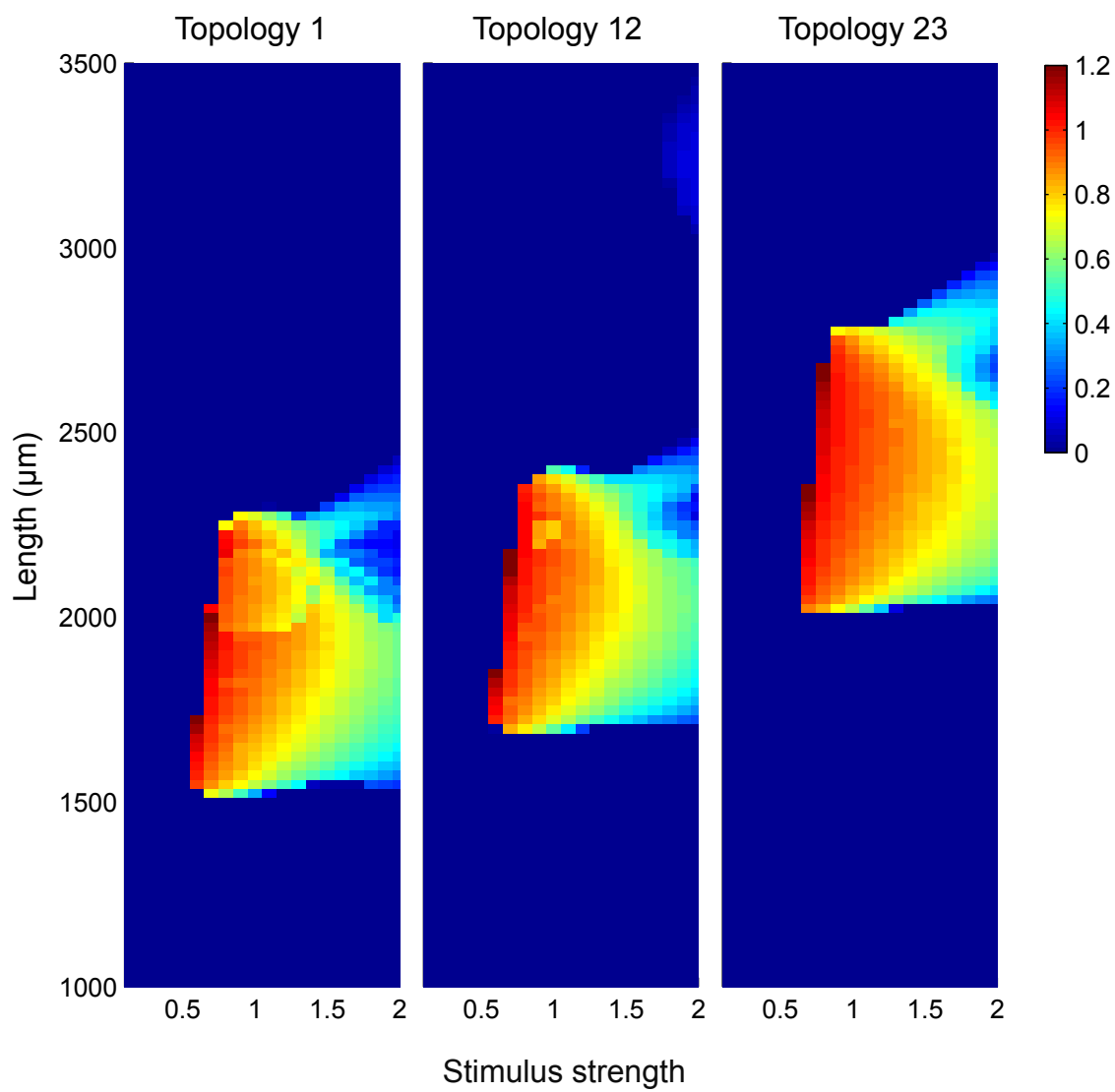

van Elburg and van Ooyen, Supplementary Figure 1

Supplement: Figure S1 — The region of burst firing is relatively insensitive to the strength of somatic stimulation. For three different tree topologies of the morphologically simplified cells (see Fig. 2; segment diameters according to Rall's power law), the degree of burst firing (color coded) is shown as a function of total dendritic length and relative stimulation strength (where 1 is our standard somatic current injection; see Methods). A doubling of the stimulation strength causes only a 5–10% shift in the position of the burst region. The weaker bursting for stronger stimuli is mainly the result of smaller interburst intervals. (0.14 MB PDF) [file pcbi.1000781.s001.pdf]

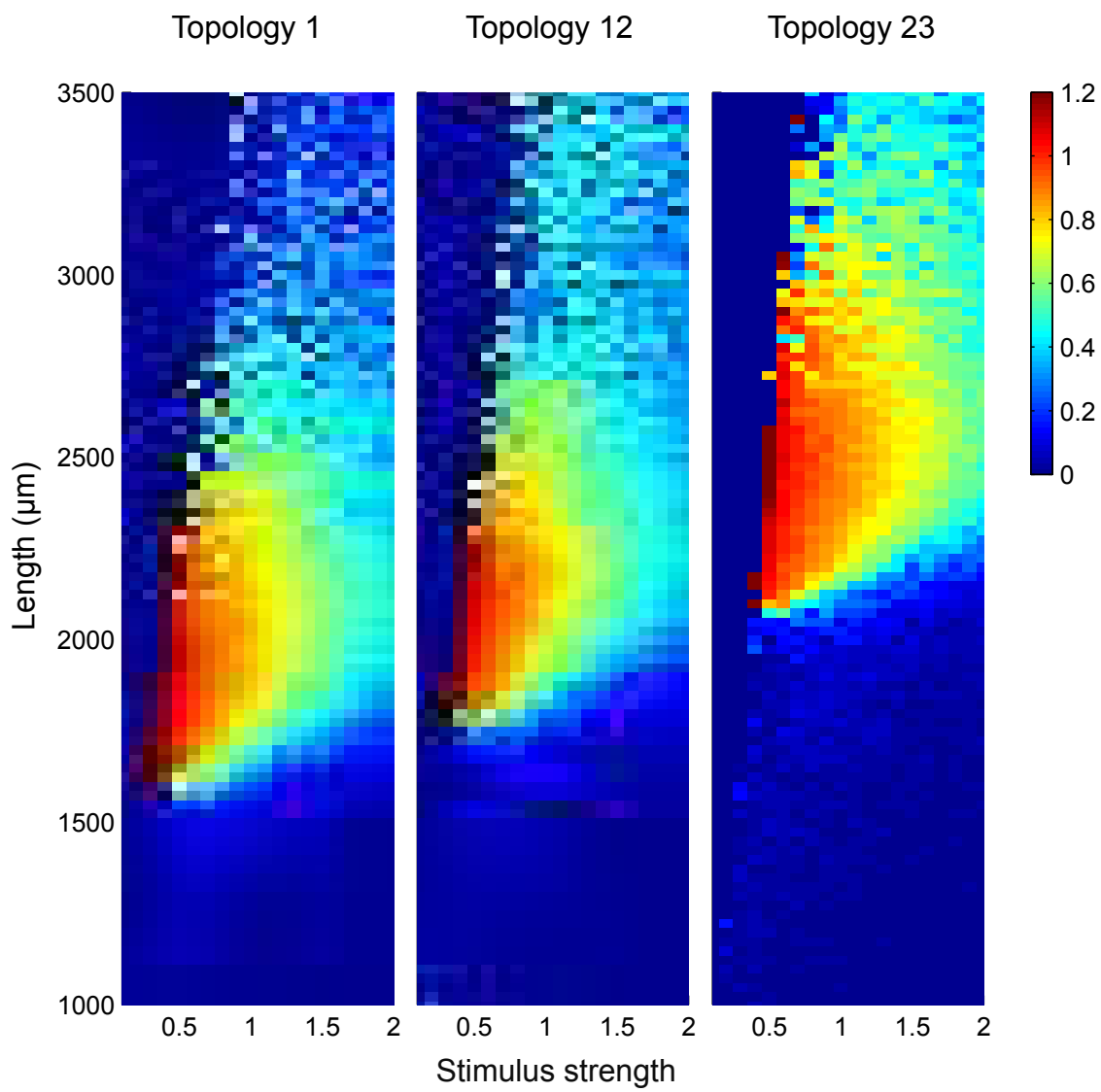

van Elburg and van Ooyen, Supplementary Figure 2

Supplement: Figure S2 — The region of burst firing is relatively insensitive to the strength of dendritic stimulation. For three different tree topologies of the morphologically simplified cells (see Fig. 2; segment diameters according to Rall's power law), the degree of burst firing (color coded) is shown as a function of total dendritic length and relative stimulation strength (where 1 is our standard synaptic peak conductance; see Methods). The weaker bursting for stronger stimuli is mainly the result of smaller interburst intervals. (0.16 MB PDF) [file pcbi.1000781.s002.pdf]

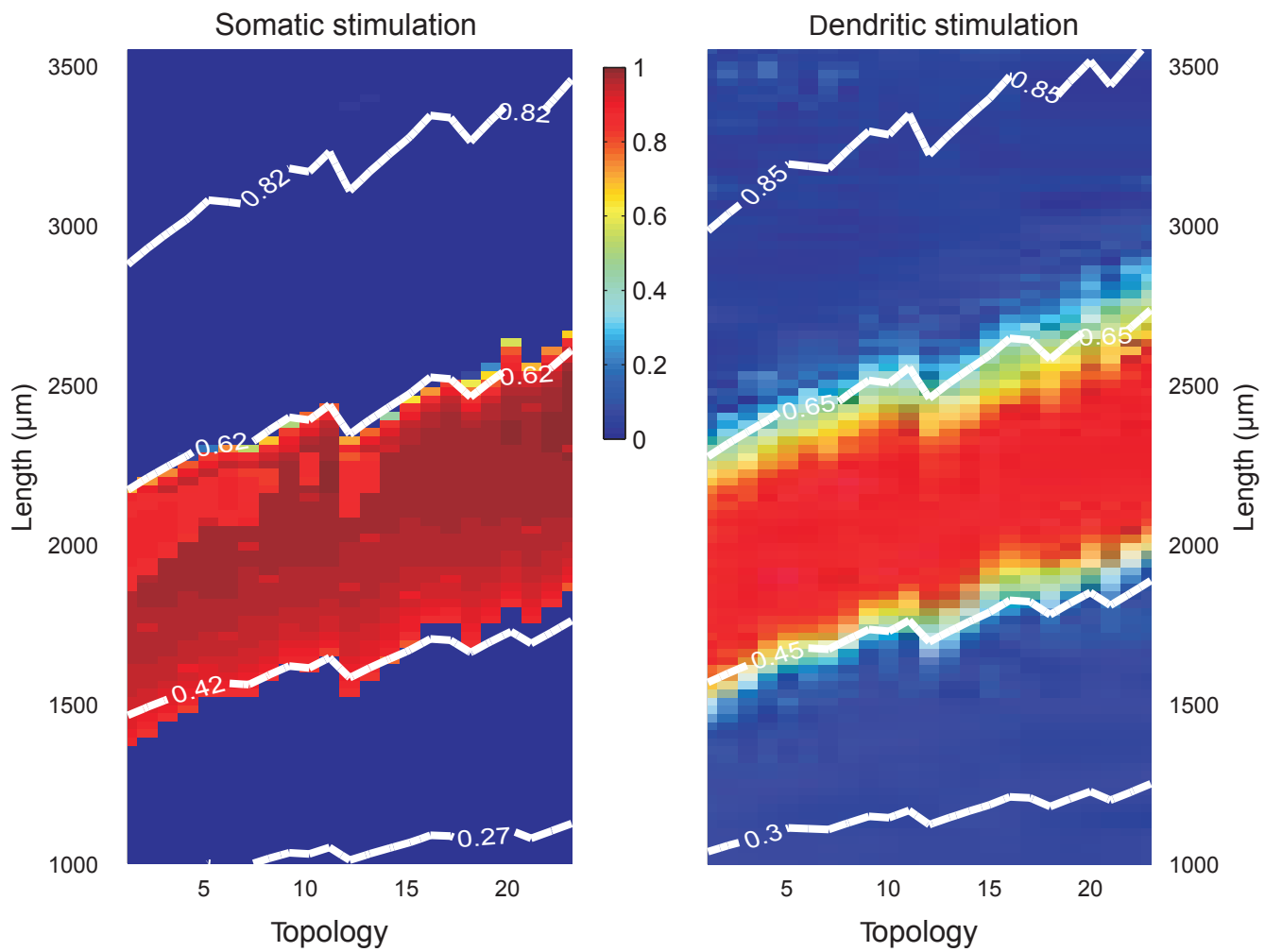

van Elburg and van Ooyen, Suppl. Figure S3.

Supplement: Figure S3 — The mean electrotonic path length correlates with the region of burst firing also when the number of ion channels is kept constant as the topology or total length of the tree is changed. To implement a constant number of ion channels, we decreased (increased) the ion channel densities (i.e., maximal conductances, expressed in pS µm∧−2) as the total surface area of the dendritic tree increased (decreased). The total dendritic surface area of the fully symmetrical tree (topology 23) at dendritic length 2500 µm was thereby taken as reference. Thus, g_x new = g_x * (surface area of the symmetrical tree at 2500 µm)/(surface area of the tree under consideration), where g_x is the standard maximal conductance as given in Methods and the index x indicates channel type. As in Fig. 10, the segment diameters of the trees obey Rall's power law. (Recall that for Rall trees, a change in tree topology also results in a different total dendritic surface area; see Methods.) The degree of burst firing (color coded) is shown for different dendritic topologies and tree sizes, together with contour lines of equal mean electrotonic path length (in units of the electrotonic length constant). (0.17 MB PDF) [file pcbi.1000781.s003.pdf]

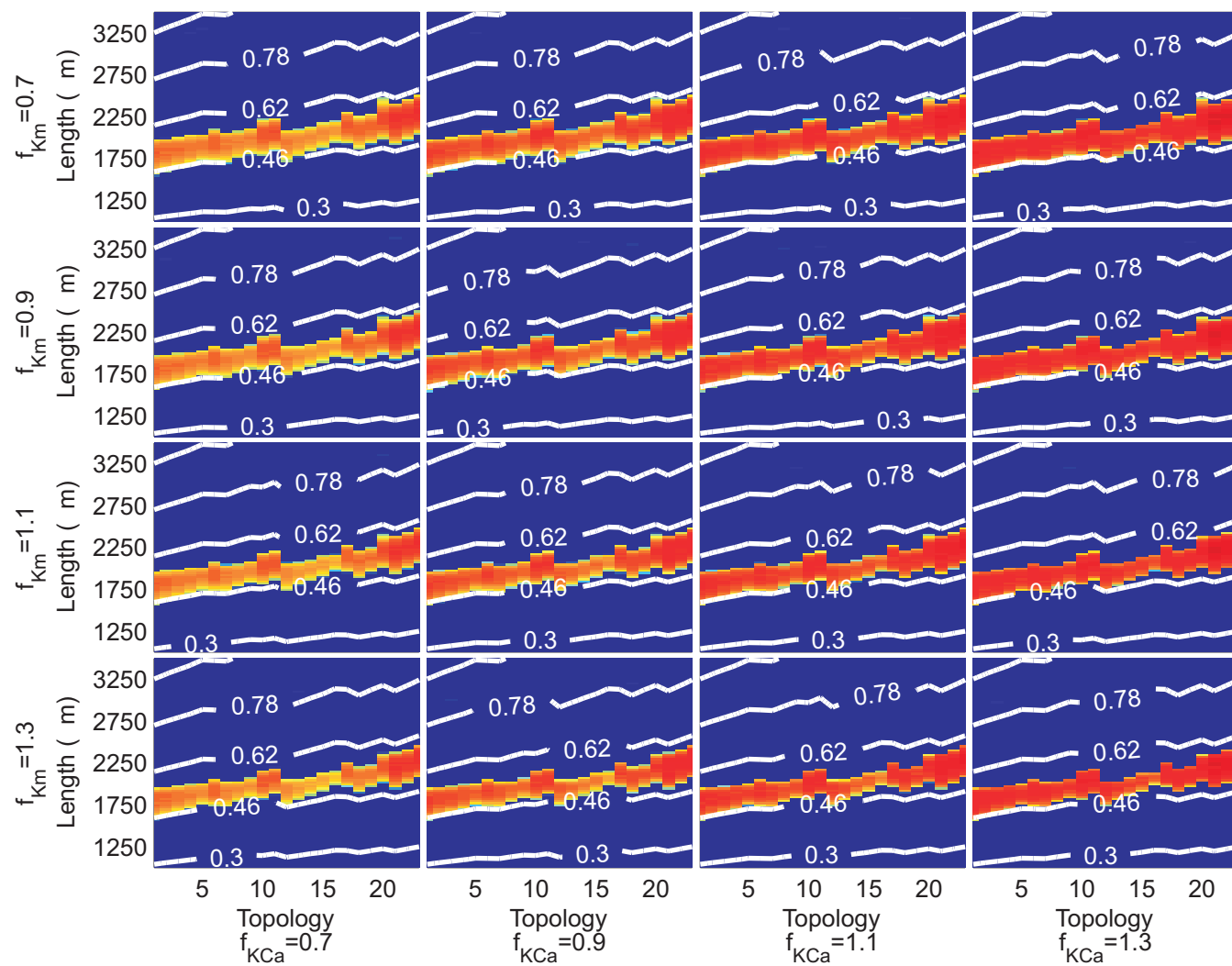

van Elburg and van Ooyen, Suppl. Figure S4.

Supplement: Figure S4 — The influence of dendritic size and topology on burst firing and the importance of mean electrotonic path length are robust to changes in ion channel densities. For a wide range of dendritic ion channel densities, the mean electrotonic path length correlates with the region of burst firing. The maximal conductance of Na is 90% of the standard value (see Methods). The maximal conductances of Km and KCa are varied. The factors f multiply the standard values of the maximal conductances. The segment diameters of the trees obey Rall's power law. The cells are stimulated by somatic stimulation. Each sub-panel, as in Figs. 9 and 10, shows the degree of burst firing (color coded) as a function of dendritic size and dendritic topology, together with contour lines of equal mean electrotonic path length (in units of the electrotonic length constant). (0.06 MB PDF) [file pcbi.1000781.s004.pdf]

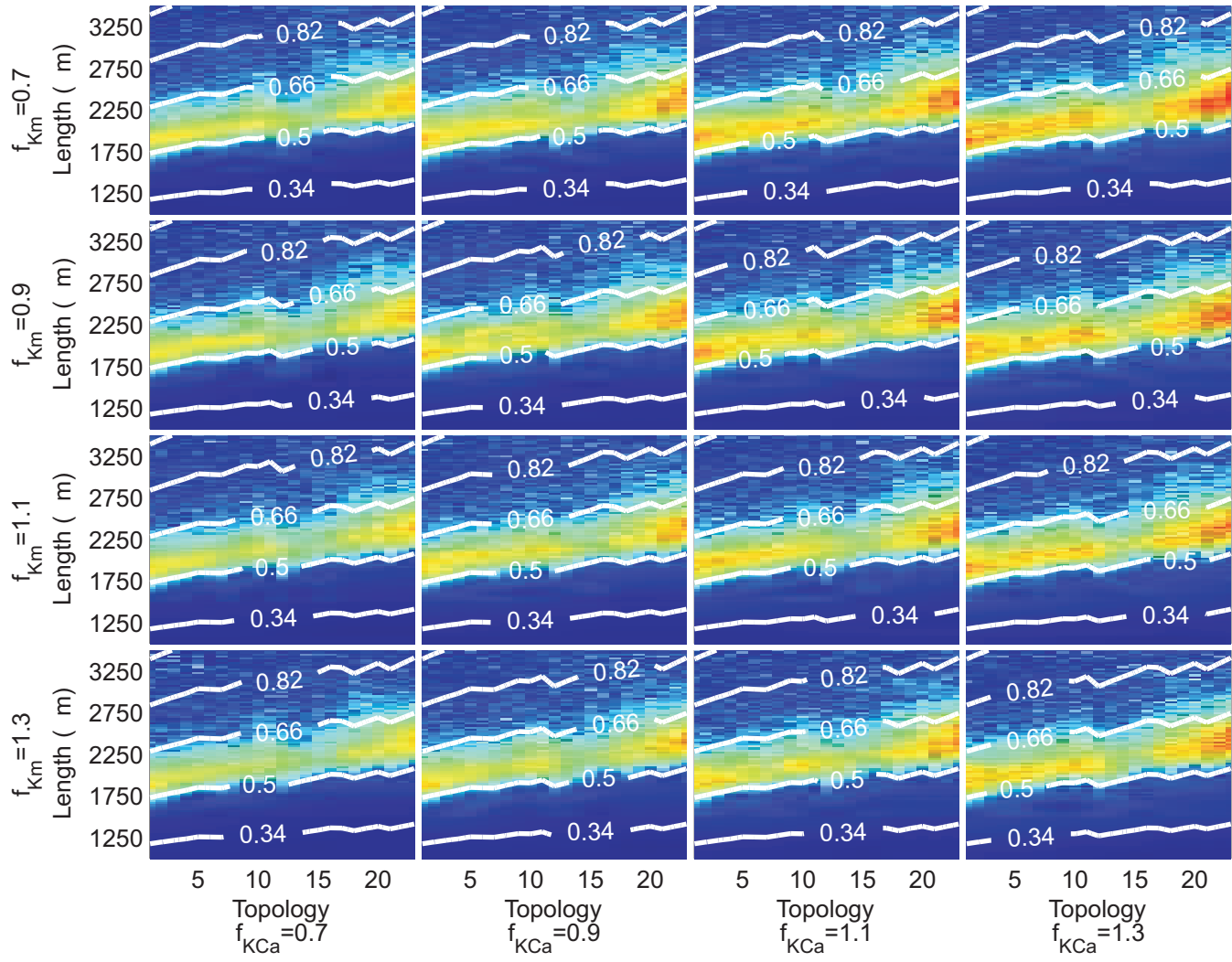

van Elburg and van Ooyen, Suppl. Figure S6.

Supplement: Figure S6 — The influence of dendritic size and topology on burst firing and the importance of mean electrotonic path length are robust to changes in ion channel densities. For a wide range of dendritic ion channel densities, the mean electrotonic path length correlates with the region of burst firing. The maximal conductance of Na is 90% of the standard value (see Methods). The maximal conductances of Km and KCa are varied. The factors f multiply the standard values of the maximal conductances. The segment diameters of the trees obey Rall's power law. The cells are stimulated by dendritic stimulation. Each sub-panel, as in Figs. 9 and 10, shows the degree of burst firing (color coded) as a function of dendritic size and dendritic topology, together with contour lines of equal mean electrotonic path length (in units of the electrotonic length constant). (0.12 MB PDF) [file pcbi.1000781.s006.pdf]

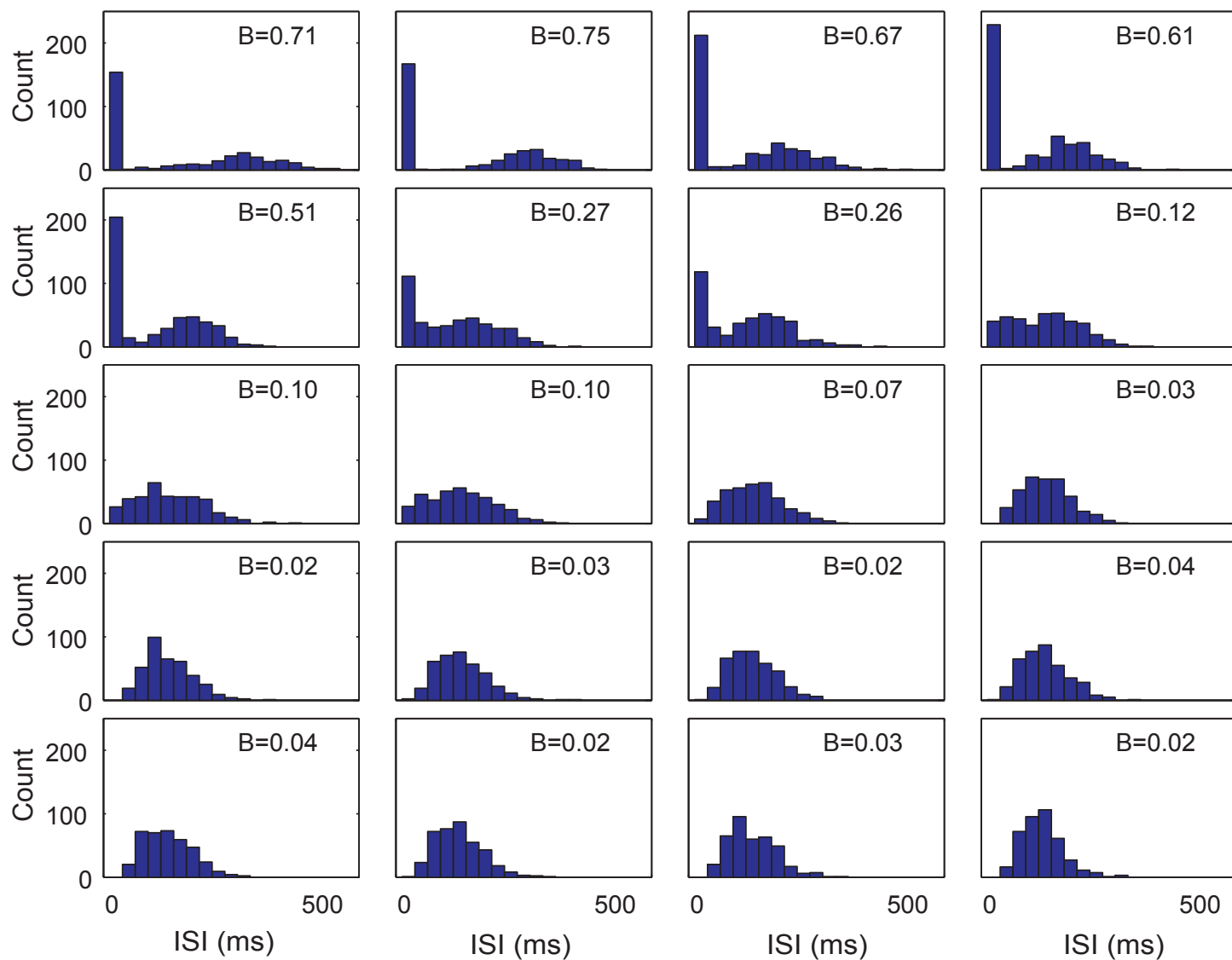

van Elburg and van Ooyen, Suppl. Figure S8.

Supplement: Figure S8 — Interspike-interval (ISI) distributions, together with burst measure values (B), in the experiment in which the total length of the pyramidal cell was gradually reduced by pruning the apical dendrite (see Fig. 3, dendritic stimulation). For every step in a single pruning sequence, the ISI distribution (bin size = 25 ms) and B value are shown. The top left graph is of the intact pyramidal cell, and every step going from left to right signifies a round of pruning (see Methods). In this experiment, B = 0.12 is already accompanied by a weakly bimodal ISI distribution, while B values larger than 0.25 are associated with marked bimodality. (0.15 MB PDF) [file pcbi.1000781.s008.pdf]
