# Supplementary material for: Impact of Dendritic Size and Dendritic Topology on Burst Firing in Pyramidal Cells
Source: PLoS Comput Biol. 2010 May 13;6(5):e1000781. doi: 10.1371/journal.pcbi.1000781 (PMC2869305; doi:10.1371/journal.pcbi.1000781)
Supplement: Figure S5 — The influence of dendritic size and topology on burst firing and the importance of mean electrotonic path length are robust to changes in ion channel densities. For a wide range of dendritic ion channel densities, the mean electrotonic path length correlates with the region of burst firing. The maximal conductance of Na is 110% of the standard value (see Methods). The maximal conductances of Km and KCa are varied. The factors f multiply the standard values of the maximal conductances. The segment diameters of the trees obey Rall's power law. The cells are stimulated by somatic stimulation. Each sub-panel, as in Figs. 9 and 10, shows the degree of burst firing (color coded) as a function of dendritic size and dendritic topology, together with contour lines of equal mean electrotonic path length (in units of the electrotonic length constant). (0.12 MB PDF) [file pcbi.1000781.s005.pdf]

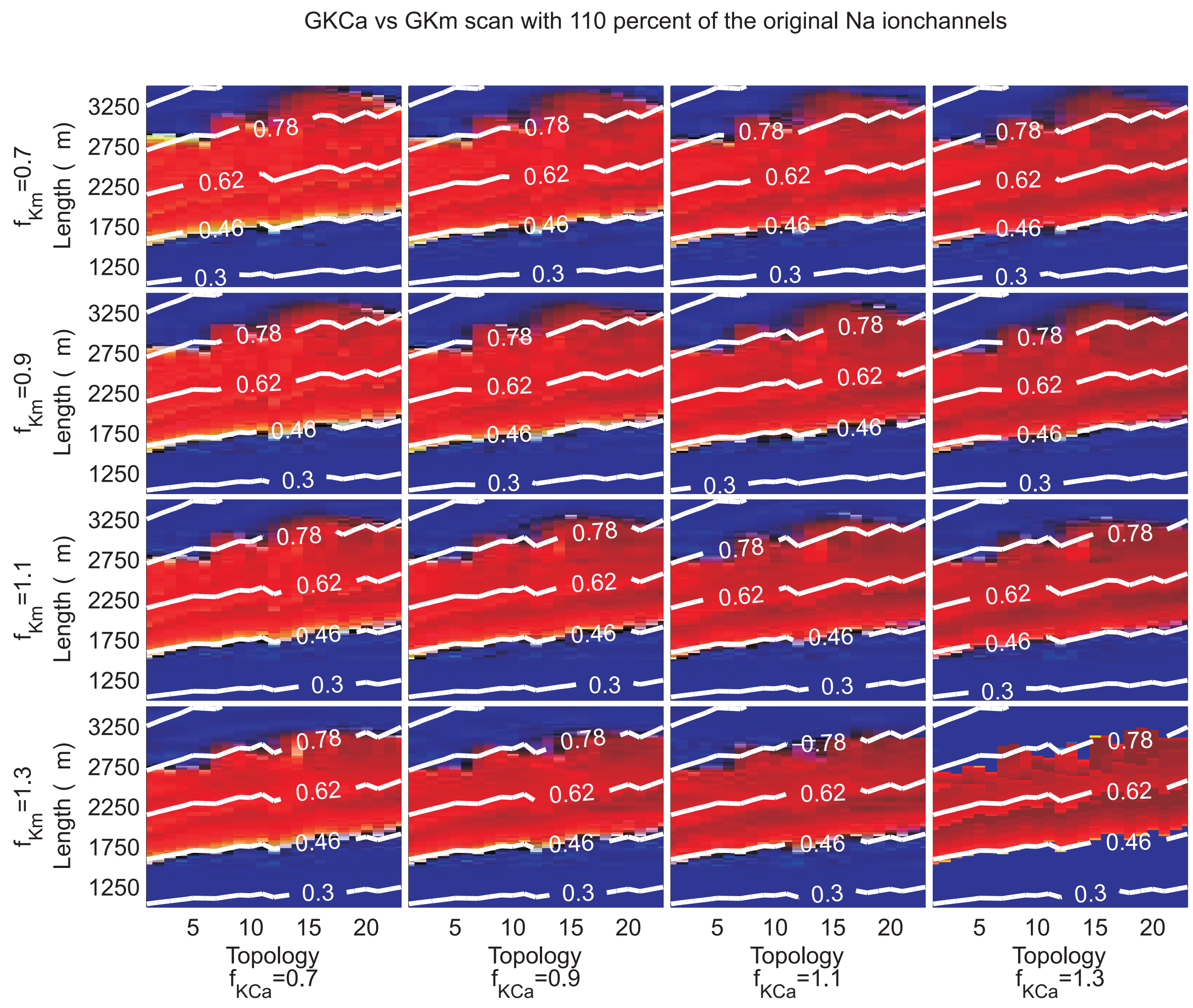

van Elburg and van Ooyen, Suppl. Figure S5.
