# Supplementary material for: Impact of Dendritic Size and Dendritic Topology on Burst Firing in Pyramidal Cells
Source: PLoS Comput Biol. 2010 May 13;6(5):e1000781. doi: 10.1371/journal.pcbi.1000781 (PMC2869305; doi:10.1371/journal.pcbi.1000781)
Supplement: Figure S7 — The influence of dendritic size and topology on burst firing and the importance of mean electrotonic path length are robust to changes in ion channel densities. For a wide range of dendritic ion channel densities, the mean electrotonic path length correlates with the region of burst firing. The maximal conductance of Na is 110% of the standard value (see Methods). The maximal conductances of Km and KCa are varied. The factors f multiply the standard values of the maximal conductances. The segment diameters of the trees obey Rall's power law. The cells are stimulated by dendritic stimulation. Each sub-panel, as in Figs. 9 and 10, shows the degree of burst firing (color coded) as a function of dendritic size and dendritic topology, together with contour lines of equal mean electrotonic path length (in units of the electrotonic length constant). Comparison of Figs. S4, S5, S6, S7 shows that although the range of dendritic sizes that exhibits burst firing may be different for different dendritic ion channel densities (with a higher density of Na channels, the dendritic length range that shows burst firing is larger; e.g., compare Figs. S4 and S5), the overall effect of dendritic size and topology on burst firing and the correlation of the region of burst firing with mean electrotonic path length is the same in all cases. Note that the value of the mean electrotonic path length where burst firing commences is the same for different combinations of ion channels densities, both under somatic stimulation (MEP = 0.46) and under dendritic stimulation (MEP = 0.50). (0.12 MB PDF) [file pcbi.1000781.s007.pdf]

GKCa vs GKm scan with 110 percent of the original Na ionchannels

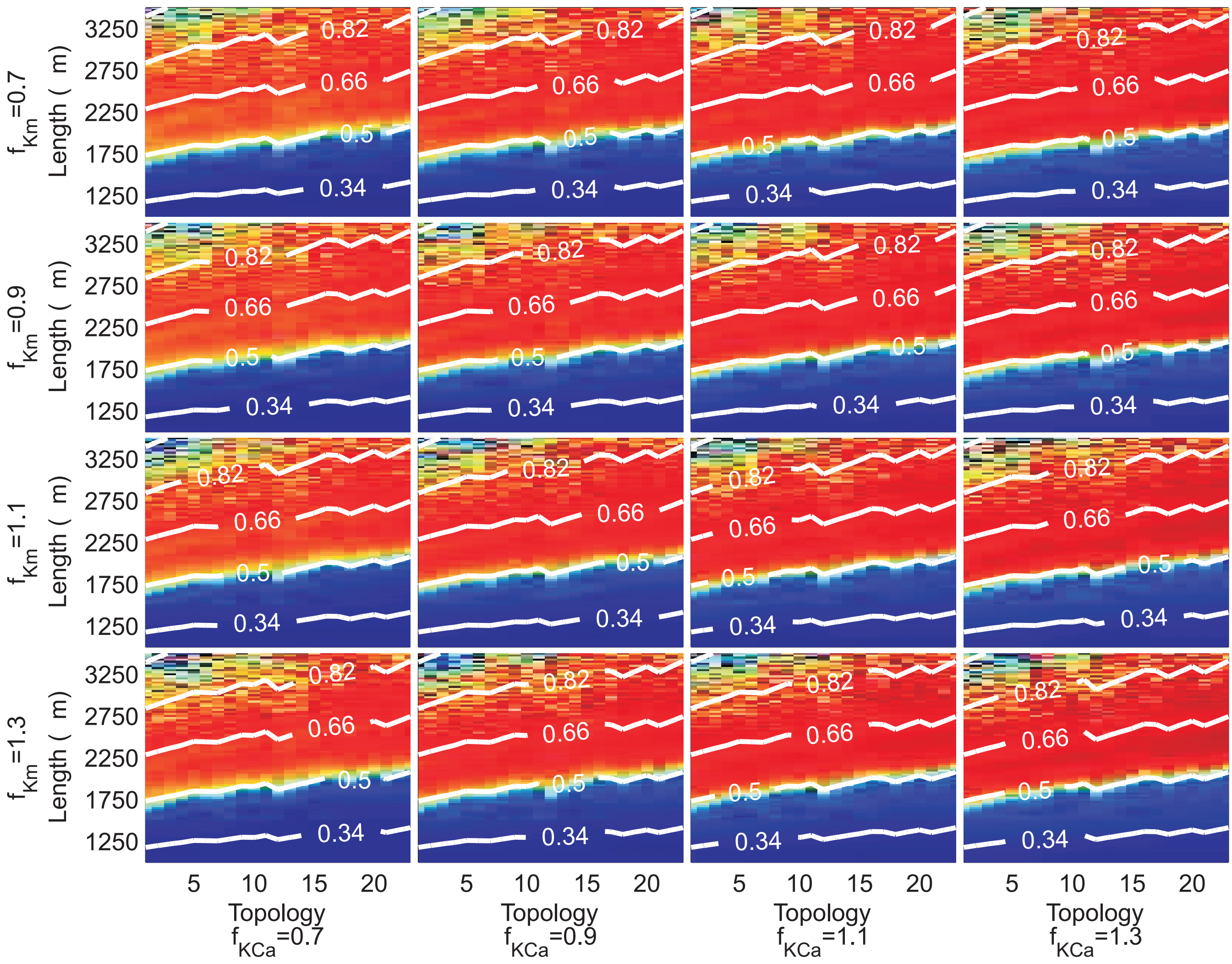

van Elburg and van Ooyen, Suppl. Figure S7.
