# Supplementary material for: Impact of Dendritic Size and Dendritic Topology on Burst Firing in Pyramidal Cells
Source: PLoS Comput Biol. 2010 May 13;6(5):e1000781. doi: 10.1371/journal.pcbi.1000781 (PMC2869305; doi:10.1371/journal.pcbi.1000781)
Supplement: Text S1 — The burst measure and the derivation of its expected value for a periodic spike train with two-spike bursts. (0.18 MB PDF) [file pcbi.1000781.s009.pdf]

Text S1: The burst measure and the derivation of its expected value for a periodic spike train with two-spike bursts

Supporting text to "Impact of dendritic size and dendritic topology on burst firing in pyramidal cells" by Ronald A. J. van Elburg and Arjen van Ooyen

To illustrate the burst measure of Van Elburg and Van Ooyen (2004) used here, consider a spike train (see Fig. A1) with spikes occurring at times t_i, where the index i runs from 1 to the total number of spikes in the spike train. The interspike interval between two successive spike is thus $t_{i+1}-t_i$, and the sum of two successive interspike intervals is $t_{i+2}-t_i$.

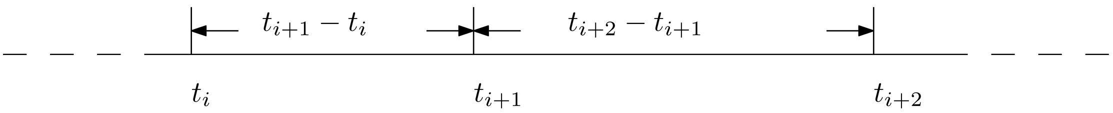

Figure A1: Spike train, where t_i denotes the time of the i-th spike in the spike train.

If a spike train consists of spikes with independent successive interspike intervals, the variance of the sum of two successive interspike intervals [$\mathrm{var}(t_{i+2}-t_i)$] is twice the variance of the single interspike intervals [$\mathrm{var}(t_{i+1}-t_i)$]. If bursting occurs, successive intervals are no longer independent, and this relation is violated. Thus, the difference between the two variances is a measure for bursting. If we divide this difference by the squared average interspike interval, a normalized burst measure (*B*) is obtained that is sensitive only to the relative sizes of the interspike intervals and not to the average interval size:

$$B = \frac{2\mathrm{var}(t_{i+1}-t_i) - \mathrm{var}(t_{i+2}-t_i)}{2E^2(t_{i+1}-t_i)} \qquad \text{(A1)}$$

where $E^2(t_{i+1}-t_i)$ means $(E(t_{i+1}-t_i))^2$, and $E(t_{i+1}-t_i)$ stands for taking the expected or average value of the interspike intervals between two successive spikes. We use eqn (A1) to quantify the extent of bursting in a spike train, taking into account all interspike intervals $t_{i+1}-t_i$ to calculate $E(t_{i+1}-t_i)$ and $\mathrm{var}(t_{i+1}-t_i)$, and all interspike

intervals $t_{i+2} - t_i$ to calculate $\mathrm{var}(t_{i+2} - t_i)$. If a spike train consists of spikes with independent successive interspike intervals (i.e., there is no bursting), $\mathrm{var}(t_{i+2} - t_i) = 2\,\mathrm{var}(t_{i+1} - t_i)$, and $B = 0$.

To further illustrate the burst measure, consider a spike train that consists of alternating long and short intervals (two-spike bursts). Suppose that the spike train was generated by alternately drawing an interval from a distribution of long T_l intervals (with mean τ_l) and a distribution of short T_s intervals (with mean τ_s) (see Fig. A2). Since this violates the independence of successive interspike intervals, we expect to find a non-zero value for our burst measure.

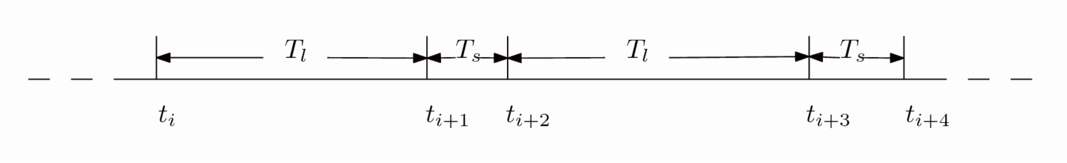

Figure A2. A spike train generated by alternately drawing interspike intervals from the distributions of long T_l and short T_s intervals, respectively.

First, recall that variances can be calculated by using $\mathrm{var}(X) = E(X^2) - E^2(X)$. The relevant means and variances for calculating the burst measure are:

$$E(t_{i+1} - t_i) = \frac{E(T_l) + E(T_s)}{2}$$

$$E\left((t_{i+1} - t_i)^2\right) = \frac{E(T_l^2) + E(T_s^2)}{2}$$

$$\begin{aligned}\mathrm{var}(t_{i+1} - t_i) &= \frac{E(T_l^2) + E(T_s^2)}{2} - \left(\frac{E(T_l) + E(T_s)}{2}\right)^2 \\ &= \frac{\mathrm{var}(T_l) + \mathrm{var}(T_s)}{2} + \left(\frac{E(T_l) - E(T_s)}{2}\right)^2\end{aligned} \qquad \text{(A2)}$$

$$\mathrm{var}(t_{i+2} - t_i) = \mathrm{var}(T_l) + \mathrm{var}(T_s)$$

Inserting these into eqn (A1) yields:

$$B = \frac{2\left(\frac{E(T_l) - E(T_s)}{2}\right)^2}{2\left(\frac{E(T_l) + E(T_s)}{2}\right)^2} \qquad \text{(A3)}$$

Using $E(T_l) = \tau_l$ and $E(T_s) = \tau_s$, we can reorganize eqn (A3) into

$$B = \left(\frac{\tau_l / \tau_s - 1}{\tau_l / \tau_s + 1}\right)^2 \qquad \text{(A4)}$$

If $\tau_l = \tau_s$, there is no bursting, and $B = 0$. The higher the ratio τ_l / τ_s of inter- to intraburst intervals, the stronger the bursting and the higher the value of B. In the limiting case $\tau_l / \tau_s \to \infty$, $B \to 1$. Fig. A3 shows B as a function of τ_l / τ_s, together with B values obtained for bursts of more than two spikes. The analog equation of eqn (A4) for bursts of more than two spikes is derived in Text S2.

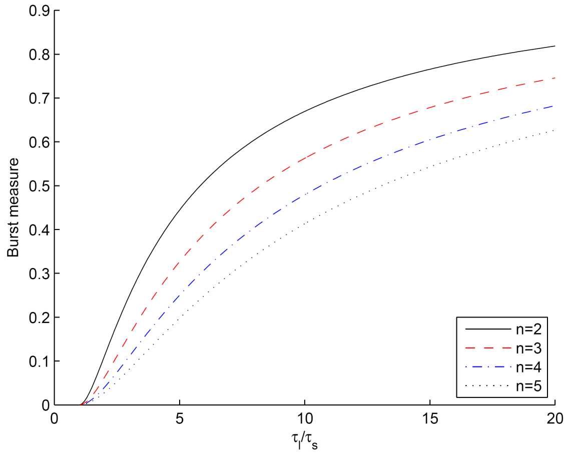

Figure A3. Burst measure as a function of the ratio of inter- to intraburst intervals for different numbers of spikes per burst (n = 2, 3, 4, 5).

Our burst measure is related to the first serial correlation coefficient (Cox and Lewis, 1966; De Kwaadsteniet, 1982). The main difference is that the serial correlation coefficient is not sensitive to the relative sizes of the interspike intervals.

Burst measures that are based on quantifying bimodality (or higher modality) in the distribution of interspike intervals detect only the occurrence of two or more clusters of spike intervals and not burst firing as such, i.e., the *correlated* occurrence of one or more short interspike intervals followed by a long interspike interval. To give an impression of the interspike-interval distributions in our data, we show in Fig. S8 the interspike intervals in the experiment in which the total length of the pyramidal cell was gradually reduced by pruning the apical dendrite (see Fig. 3).

References

Cox DR, Lewis PAW. *The Statistical Analysis of Series of Events*, Methuen & Co Ltd, London, 1966.

De Kwaadsteniet JW (1982) Statistical analysis and stochastic modelling of neuronal spike-train activity. Math. Biosci. 60: 17-71.

Van Elburg RAJ, Van Ooyen A (2004) A new measure for bursting. Neurocomputing. 58-60: 497-502.
