# Supplementary material for: Impact of Dendritic Size and Dendritic Topology on Burst Firing in Pyramidal Cells
Source: PLoS Comput Biol. 2010 May 13;6(5):e1000781. doi: 10.1371/journal.pcbi.1000781 (PMC2869305; doi:10.1371/journal.pcbi.1000781)
Supplement: Text S2 — Burst firing described as a semi-Markov process and generalization of the burst measure to n-spike bursts (0.13 MB PDF) [file pcbi.1000781.s010.pdf]

Text S2: Burst firing described as a semi-Markov process and generalization of the burst measure to *n*-spike bursts

Supporting text to "Impact of dendritic size and dendritic topology on burst firing in pyramidal cells" by Ronald A. J. van Elburg and Arjen van Ooyen

The scenario introduced in Text S1, with alternating long and short interspike intervals (i.e., a two-spike burst), is an example of an alternating renewal process. Alternating renewal processes can also be described as semi-Markov processes. This semi-Markov process description will be used here because it allows us to generalize the theoretical burst measure calculation to trains of *n*-spike bursts.

Again we first consider a spike train consisting of alternating long and short intervals (two-spike burst). The semi-Markov process describing this simple alternating scenario has two states corresponding to the first spike d_1 and the second spike d_2 of a two-spike burst. The transition probabilities of going from d_1 to d_2 and vice versa are given by $p_{d_2d_1} = p_s$ and $p_{d_1d_2} = p_l$, respectively. The transition probabilities from a state to the next event in the same state are given by $p_{d_1d_1} = 1 - p_s$ and $p_{d_2d_2} = 1 - p_l$. The transition times between the states are described by random variables $T_{d_id_j}$. Since in trains of *n*-spike bursts, transitions from a state to a next event in the same state do not occur and only transitions between different states are defined, we only give the mean values associated with these latter transitions, $E(T_{d_2d_1}) = \tau_s$ and $E(T_{d_1d_2}) = \tau_l$. The equilibrium distribution describing the probabilities with which an arbitrary spike belongs to one of the states associated with this two-state semi-Markov chain is given by (Cox and Lewis, 1966):

$$\pi_{d_1} = \frac{p_l}{p_s + p_l}, \quad \pi_{d_2} = \frac{p_s}{p_s + p_l} \qquad \text{(B1)}$$

For an alternating renewal process with $p_s = p_l = 1$ we therefore obtain $\pi_{d_1} = \pi_{d_2} = 1/2$.

We will now extend the two-state semi-Markov process by considering a semi-Markov process with an arbitrary number of states labeled by $d_1, d_2, \cdots, d_n$,

labels that refer to the first, the second, until the *n*-th spike in an *n*-spike burst. Instead of writing explicit expressions involving these labels, we introduce the state variables $\alpha, \beta, \gamma, \varepsilon$, which take on values in this set of labels. We will use these to express our burst measure in terms of the transition probabilities $p_{\alpha\beta}$, equilibrium distribution $\{\pi_\alpha\}$ and moments of the distributions of the transition times $T_{\alpha\beta}$. Now, an expectation value involving functions of the interspike interval duration, i.e., $E(f(t_{i+1}-t_i))$, can be calculated by weighing the expectation values of the individual stochastic variables by the probability of their occurrence. The probability that the stochast $f(T_{\alpha\beta})$ is realized is given by the probability π_β of finding the system in state β multiplied by the probability $p_{\alpha\beta}$ that starting from state β we have a transition to state α. For the average interspike interval and interspike interval squared we therefore obtain,

$$E(t_{i+1}-t_i) = \sum_{\alpha\beta} p_{\alpha\beta}\pi_\beta E(T_{\alpha\beta}),$$
$$E\left((t_{i+1}-t_i)^2\right) = \sum_{\alpha\beta} p_{\alpha\beta}\pi_\beta E\left(T_{\alpha\beta}^2\right)$$

To calculate expectation values involving functions of the duration of two subsequent interspike intervals, i.e., $E(f(t_{i+2}-t_i))$, we again need to weigh the appropriate combination of stochastic variables by their probability of occurrence. The probability that the stochast $f(T_{\alpha\beta}+T_{\beta\gamma})$ is realized is given by the probability π_γ of finding the system in state γ multiplied by the probability $p_{\beta\gamma}$ that starting from state γ we have a transition to state β further multiplied by the probability $p_{\alpha\beta}$ that from this state β we have a transition to state α. Thus,

$$
\begin{aligned}
E(t_{i+2}-t_i) &= \sum_{\alpha\beta\gamma} p_{\alpha\beta}p_{\beta\gamma}\pi_\gamma E(T_{\alpha\beta}+T_{\beta\gamma}) \\
&= \sum_{\alpha\beta\gamma} p_{\alpha\beta}p_{\beta\gamma}\pi_\gamma E(T_{\alpha\beta}) + \sum_{\alpha\beta\gamma} p_{\alpha\beta}p_{\beta\gamma}\pi_\gamma E(T_{\beta\gamma}) \\
&= \sum_{\alpha\beta} p_{\alpha\beta}\pi_\beta E(T_{\alpha\beta}) + \sum_{\beta\gamma} p_{\beta\gamma}\pi_\gamma E(T_{\beta\gamma}) \\
&= 2\sum_{\alpha\beta} p_{\alpha\beta}\pi_\beta E(T_{\alpha\beta}), \\
E\left((t_{i+2}-t_i)^2\right) &= \sum_{\alpha\beta\gamma} p_{\alpha\beta}p_{\beta\gamma}\pi_\gamma E\left((T_{\alpha\beta}+T_{\beta\gamma})^2\right) \\
&= \sum_{\alpha\beta\gamma} p_{\alpha\beta}p_{\beta\gamma}\pi_\gamma \left(\mathrm{var}(T_{\alpha\beta}+T_{\beta\gamma}) + E^2(T_{\alpha\beta}+T_{\beta\gamma})\right) \\
&= \sum_{\alpha\beta\gamma} p_{\alpha\beta}p_{\beta\gamma}\pi_\gamma \left(\mathrm{var}(T_{\alpha\beta}) + \mathrm{var}(T_{\beta\gamma}) + E^2(T_{\alpha\beta}+T_{\beta\gamma})\right) \\
&= 2\sum_{\alpha\beta} p_{\alpha\beta}\pi_\beta \,\mathrm{var}(T_{\alpha\beta}) + 2\sum_{\alpha\beta} p_{\alpha\beta}\pi_\beta E^2(T_{\alpha\beta}) + 2\sum_{\alpha\beta\gamma} p_{\alpha\beta}p_{\beta\gamma}\pi_\gamma E(T_{\alpha\beta})E(T_{\beta\gamma})
\end{aligned}
\tag{B2}
$$

In these equations, we have expressed all expectation values in moments of the transition time distributions using that the variance of the sum of two independent stochastic variables is the sum of the variances of the two stochastic variables. We insert these expectation values in our burst measure (Van Elburg and Van Ooyen, 2004)

$$
B = \frac{2\mathrm{var}(t_{i+1}-t_i) - \mathrm{var}(t_{i+2}-t_i)}{2E^2(t_{i+1}-t_i)} \tag{B3}
$$

which yields, using that $\mathrm{var}(X) = E(X^2) - E^2(X)$:

$$
B = \left(1 - \frac{\sum_{\alpha\beta\gamma} p_{\alpha\beta}p_{\beta\gamma}\pi_\gamma E(T_{\alpha\beta})E(T_{\beta\gamma})}{\left(\sum_{\alpha\beta} p_{\alpha\beta}\pi_\beta E(T_{\alpha\beta})\right)^2} \right) \tag{B4}
$$

Substituting into this general expression the proper values for the two-spike burst spike train, i.e. $\pi_{d_1} = \pi_{d_2} = 1/2$, $p_{d_2 d_1} = p_{d_1 d_2} = 1, p_{d_2 d_2} = p_{d_1 d_1} = 0$, $E(T_{d_2 d_1}) = \tau_s$ and $E(T_{d_1 d_2}) = \tau_l$, we obtain

$$B = 1 - \frac{\frac{1}{2}(\tau_s\tau_l + \tau_l\tau_s)}{\left(\frac{1}{2}(\tau_s + \tau_l)\right)^2} = \left(\frac{\frac{\tau_l}{\tau_s} - 1}{\frac{\tau_l}{\tau_s} + 1}\right)^2 \tag{B5}$$

which is equal to the result we obtained from our direct analysis in Text S1 [eqn (A4)].

As mentioned, the strength of this analysis is that it can be carried over to a train of *n*-spike bursts (with $n \geq 2$) without change. For a train of *n*-spike bursts we obtain for the equilibrium distribution $\pi_\alpha = \frac{1}{n}, \alpha = d_1, \cdots, d_n$. We further assume, for the sake of this analysis, that within a burst the expected interspike intervals are the same, i.e., $E\left(T_{d_{j+1}d_j}\right) = \tau_s$ for $j = 1, \cdots, n-1$; and for the long interval we set $E\left(T_{d_1 d_n}\right) = \tau_l$. Under these assumptions, we find that out of the *n* possible states, *n*-2 are followed by two successive short intervals, one by a short interval followed by a long interval, and one by a long interval followed by a short interval . Using equation (B4) we now obtain

$$B = 1 - \frac{\frac{1}{n}\left((n-2)\tau_s^2 + 2\tau_s\tau_l\right)}{\left(\frac{(n-1)\tau_s + \tau_l}{n}\right)^2} \tag{B6}$$

which can be rewritten as

$$B = \left(\frac{\frac{\tau_l}{\tau_s} - 1}{\frac{\tau_l}{\tau_s} + (n-1)}\right)^2 \tag{B7}$$

Note that for $n = 2$ (two-spike burst), eqn (B5) is recovered. Equation (B7) shows—as could already be seen in Fig. A3, which is based on eqn (B7)—that for a higher number of spikes in a burst, a larger interburst separation is needed to obtain the same value of the burst measure.

There is a subtlety involved in using methods for semi-Markov processes for a train of *n*-spike bursts. The semi-Markov process description seems to necessitate averaging over different possible states. However, it is clear that in a single (infinite) realization of a train of *n*-spike bursts, the system will visit all states with a relative frequency corresponding to the probabilities derived from the semi-Markov process analysis. From this observation it follows that averaging over a single realization of such a spike train will yield the same result as averaging over the different possible states. Provided the spike train is of sufficient duration, it is therefore allowed to compare the theoretically calculated burst measure with the burst measure calculated on a single spike train without the need to build an ensemble average.

References

Cox DR, Lewis PAW. *The Statistical Analysis of Series of Events*, Methuen & Co Ltd, London, 1966.

Van Elburg RAJ, Van Ooyen A (2004) A new measure for bursting. Neurocomputing. 58-60: 497-502.
